# *In vivo* single-molecule imaging identifies altered dynamics of calcium channels in dystrophin-mutant *C. elegans*

**DOI:** 10.1038/ncomms5974

**Published:** 2014-09-18

**Authors:** Hong Zhan, Ramunas Stanciauskas, Christian Stigloher, Kevin K. Dizon, Maelle Jospin, Jean-Louis Bessereau, Fabien Pinaud

**Affiliations:** 1 University Claude Bernard Lyon 1, CGphiMC UMR CNRS 5534, Villeurbanne 69622, France; 2 Department of Biological Sciences, Dana and David Dornsife College of Letters, Arts and Sciences, University of Southern California, Los Angeles, California 90089, USA; 3 Division of Electron Microscopy, Biocenter of the University of Würzburg, Am Hubland, 97074 Würzburg, Germany; 4 Department of Chemistry, Dana and David Dornsife College of Letters, Arts and Sciences, University of Southern California, Los Angeles, California 90089, USA; 5 Department of Physics and Astronomy, Dana and David Dornsife College of Letters, Arts and Sciences, University of Southern California, 1050 Childs way, Los Angeles, California 90089, USA

## Abstract

Single-molecule (SM) fluorescence microscopy allows the imaging of biomolecules in cultured cells with a precision of a few nanometres but has yet to be implemented in living adult animals. Here we used split-GFP (green fluorescent protein) fusions and complementation-activated light microscopy (CALM) for subresolution imaging of individual membrane proteins in live *Caenorhabditis elegans (C. elegans)*. *In vivo* tissue-specific SM tracking of transmembrane CD4 and voltage-dependent Ca^2+^ channels (VDCC) was achieved with a precision of 30 nm within neuromuscular synapses and at the surface of muscle cells in normal and dystrophin-mutant worms. Through diffusion analyses, we reveal that dystrophin is involved in modulating the confinement of VDCC within sarcolemmal membrane nanodomains in response to varying tonus of *C. elegans* body-wall muscles. CALM expands the applications of SM imaging techniques beyond the petri dish and opens the possibility to explore the molecular basis of homeostatic and pathological cellular processes with subresolution precision, directly in live animals.

## Abstract (web summary)

Single molecule fluorescence microscopy is a powerful technique to study protein dynamics in cells, but it has not been applied to adult animals. The authors use complementation-activated light microscopy in *C. elegans* to discover that dystrophin regulates the diffusion properties of voltage-dependent calcium ion channels at the surface of body-wall muscle cells.

## Graphical abstract

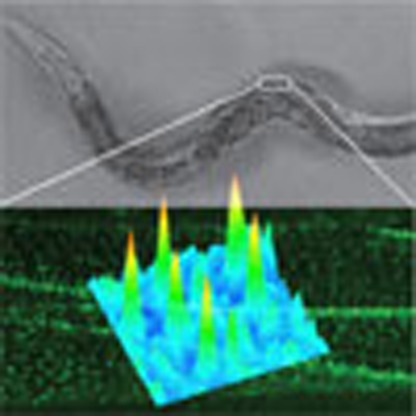

I*n vivo* fluorescence optical imaging is a powerful method to characterize biological processes in live animal tissues. Yet, the spatial precision at which these processes can be studied is constrained by the fact that lateral resolutions in conventional optical imaging are diffraction limited to ~200 nm, a length scale well above the nanometre-range interactions of most biomolecules. In recent years, single-molecule (SM) fluorescence microscopy techniques have provided means to study the mobility and the function of biomolecules beyond this resolution limit and with a precision of a few nanometres in cultured cells^1^. Extending these techniques to live animal imaging could open new avenues to examine the nanoscale behaviours of signaling molecules under homeostatic control within live tissues and during various developmental or pathogenic stages. Despite previous efforts to track individual proteins and study protein–protein interactions in embryos and early larval stages of zebrafish^2^,^3^,^4^ and *C. elegans* nematodes^5^,^6^, SM fluorescence imaging in intact adult animals remains highly challenging. *In vivo*, tissue autofluorescence often results in poor signal-to-noise ratio when attempting to detect individual fluorophores. This issue can be addressed using high-contrast imaging techniques such as highly inclined and laminated optical sheet excitation (HILO)^7^ or selective plane illumination microscopy^8^,^9^; however, advanced labelling methods are still required for site-specific SM imaging. In particular, maintaining a nanomolar SM detection regimen at subcellular locations in a live animal necessitates site-directed and stochiometric labelling or detection of a few biomolecules, despite their endogenous expression at levels generally well above those required for SM studies.

SM detection regimes can be achieved using traditional fluorescent protein (FP) fusions expressed *in vivo*; however, it generally requires extensive and potentially toxic photobleaching or alteration of protein expressions^6^. While this can be avoided using fusions to photoactivable FPs^10^, the photoactivation of a limited number of molecules at specific organelles within live animal tissues is complicated by the fact that FP mature before the fusions reach their final cellular destination. An alternative is to label biomolecules post-translationally and in a controllable manner by intravital injection of synthetic fluorescence tags. However, most labelling strategies demand micromolar concentrations of probes^11^, which generally results in high levels of nonspecific binding to tissues that cannot be washed-off. Moreover, residual non-targeted probes circulating in the body of injected animals further hinder SM imaging.

Recently, we introduced complementation-activated light microscopy (CALM), a SM technique that allows nanometre-precision imaging of individual biomolecules in living cells, independently of their expression levels, at micromolar concentration of fluorescent probes, and without the need to wash excess probes^12^. In CALM, proteins of interest are fused to a dark, non-mature split-GFP (green fluorescent protein) that lacks a 16 amino-acid β-strand. They are then fluorescently activated by irreversible binding and complementation with synthetic peptide sequences encoding the missing GFP β-strand (M3 peptides). These small synthetic M3 peptides permit site-specific imaging and tracking of individual fusion proteins by direct detection of activated split-GFP (dCALM) or by single pair Förster resonance energy transfer with fluorescently labelled M3 peptides (CALM-spFRET)^12^. Here we applied CALM to tissue-specific imaging and SM tracking of CD4 and voltage-dependent Ca^2+^ channel (VDCC) membrane proteins in live adult *C. elegans*.

In *C. elegans*, VDCCs are located in the membrane of excitable cells where they mediate the influx of extracellular Ca^2+^ in response to depolarizing signals. They play an essential role for electrochemical coupling in motor neurons by triggering neurotransmitter release^13^ and for excitation–contraction coupling in body-wall muscle cells where they elicit action potentials^14^. A dysregulation of VDCC activity in dystrophin-mutant *C. elegans* has recently been proposed to be a key factor for the genesis of muscular dystrophy^15^; however, the channels’ location and membrane dynamics have not been studied in living animals. Here we imaged and tracked individual VDCC at the sarcolemma of muscle cells and within neuromuscular synapses of normal and dystrophin-mutant *C. elegan*s, using CALM. We demonstrate that dystrophin is a load-bearing element and an adaptive tension transducer involved in modulating the nanoscale confinement of VDCC at the sarcolemma in response to changes in muscle tonus. Our results illustrate that SM imaging and tracking by CALM allows a phenotyping of diseases such as muscular dystrophy with subresolution precision directly in intact animals.

## Results

### Live tissue fluorescence imaging by CALM in *C. elegans*

**Figure 1:**
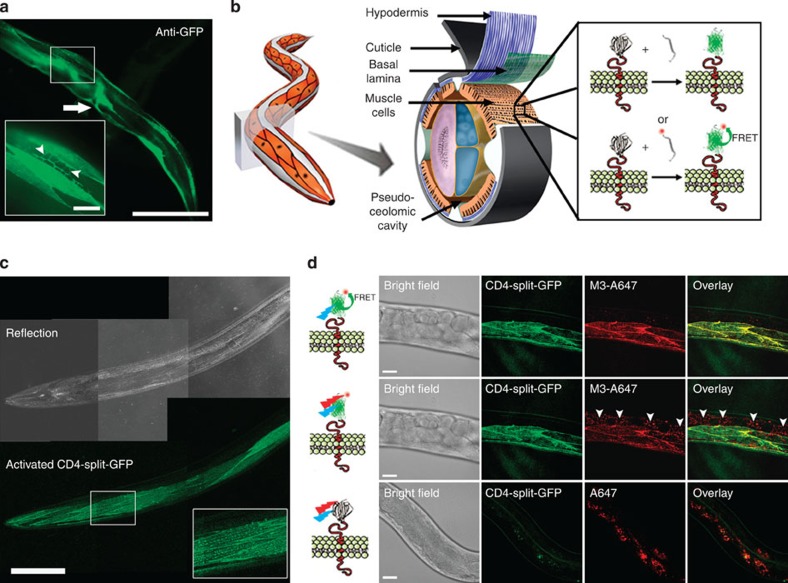
Ensemble CALM imaging of CD4-split-GFP in *C. elegans*. (**a**) GFP immunofluorescence staining of *C. elegans* expressing CD4-split-GFP under a muscle-specific promoter. CD4-split-GFP is detected in body-wall muscle somas, muscle arms (arrowheads, inset) and in vulval muscles (arrow). Scale bars: 150 and 25 μm (inset). (**b**) Schematic of dCALM and CALM-spFRET by microinjection of synthetic M3 peptides in the pseudoceolomic cavity of worms and activation of transmembrane CD4-split-GFP at muscle cell membranes. Adapted with permission from ref. 62 and from Z. F. Altun and D. H. Hall (wormatlas.org). (**c**) Live worm dCALM confocal imaging of activated CD4-split-GFP at the surface of body-wall muscles. Scale bar: 100 μm. (**d**) CALM-spFRET confocal imaging of CD4-split-GFP in live *C. elegans.* Single excitation at 488 nm allows muscle-specific near-infrared imaging of CD4-split-GFP (top) by eliminating interferences from nonspecifically bound peptides detected by dual 488 and 647 nm excitation (middle, arrowheads). No complementation is observed when injecting worms with the unconjugated A647 dye (bottom). Scale bars: 25 μm.

To explore the applications of dCALM and CALM-spFRET for live tissue imaging, we first used a *C. elegans* strain expressing a transmembrane CD4-split-GFP fusion under the muscle-specific *myo-3* promoter^16^ (Fig. 1a). Split-GFP complementary M3 peptides were microinjected at high concentration in the fluid-filled pseudocoelomic cavity of live worms to allow their distribution to all organs and the activation of CD4-split-GFP on muscle cells (Fig. 1b and Supplementary Movie 1). Injected M3 peptides rapidly triggered the specific GFP activation of CD4-split-GFP on body-wall and vulval muscles (Fig. 1c and Supplementary Fig. 1). When worms were injected with M3 peptides conjugated to an Alexa 647 fluorophore (M3-A647)-specific CALM-spFRET imaging of CD4-split-GFP could also be performed at 680 nm using only 488 nm excitation (Fig. 1d). The large-excitation Stokes shift afforded by CALM-spFRET prevented a direct excitation of residual M3-A647 nonspecifically bound to other tissues and allowed muscle-specific near-infrared imaging of CD4-split-GFP in living worms (Fig. 1d). FRET signals on muscle cells were exclusively triggered by 1:1 stochiometric and irreversible binding of M3-A647 to CD4-split-GFP, as confirmed by *in vitro* fluorescence correlation spectroscopy (FCS) with recombinant split-GFP at picomolar concentrations (Supplementary Fig. 2). Neither complementation nor FRET was observed in transgenic worms injected with unconjugated A647 (Fig. 1d).

### Single-molecule tracking of CD4-split-GFP on muscle cells

**Figure 2:**
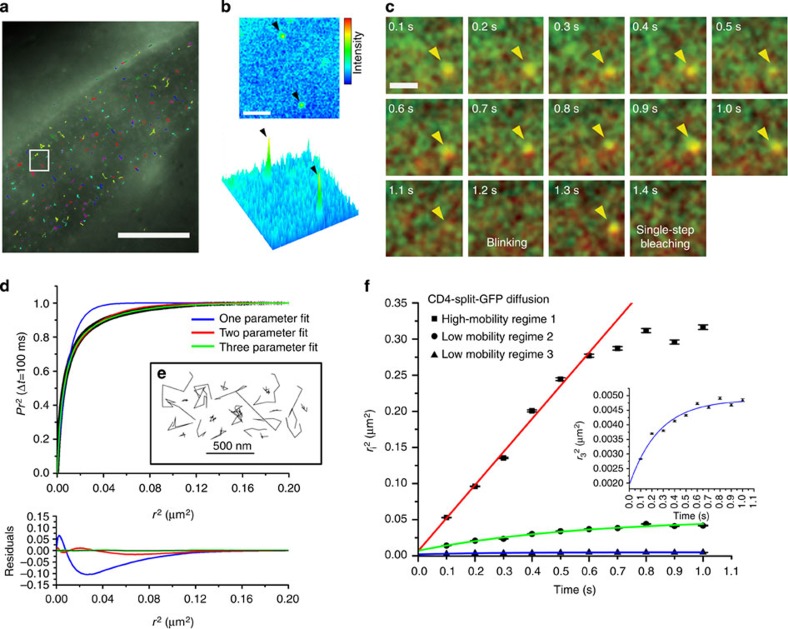
Single-molecule tracking of CD4-split-GFP on muscle cells by dCALM and CALM-spFRET. (**a**) Individual activated CD4-split-GFP trajectories at the plasma membrane of a dorsal muscle cell. Scale bar: 20 μm. (**b**) Single frame image from the white square in **a** showing a 2D and 3D representation of the GFP signal for two individual activated CD4-split-GFP (arrowheads). Scale bar: 2 μm. (**c**) CALM-spFRET imaging of an individual CD4-split-GFP activated by an M3-A647 peptide (yellow arrow head). Individual time frames show the overlay of GFP and A647 channels during 488 nm excitation only. Blinking (1.2 s) and single-step photobleaching (1.4 s) are observed. Diffraction-limited spots are intentionally expanded to facilitate visualization. Scale bar: 1 μm. (**d**) Analysis of CD4-split-GFP diffusion by PDSD for time lag Δ*t*=100 ms. The distribution is best described by a three-parameter fit indicative of three regimes of diffusion for CD4-split-GFP at the sarcolemma. (**e**) Representative example of individual CD4-split-GFP trajectories. (**f**) Diffusion coefficient analysis of the three diffusion regimes for 6193 CD4-split-GFP on 21 muscle cells from five worms. The high-mobility regime is best fitted with a free diffusion model at short times (red fit), while both low-mobility regimes show confined diffusion (green and blue fits). Inset: Close up on low-mobility regime 3. Error bars represent the s.e. of each mean.

In CALM, the simple titration of complementary M3 peptides in extracellular fluids permits a controlled SM imaging of split-GFP fusion proteins specifically at the outer plasma membrane of cells because the peptides do not activate intracellular split-GFP if they are not injected directly in the cytoplasm^12^. To control the activation rate of membrane CD4-split-GFP and achieve continuous SM detection, we injected worms with low concentrations of M3 peptides. SM dCALM imaging was performed at the interface between the basal lamina and the sarcolemma of body-wall muscles using 488 nm HILO excitation. Under these conditions, activated CD4-split-GFP appearing as individual GFP diffraction-limited spots (Fig. 2a,b and Supplementary Movie 2) could be tracked by two-dimensional (2D) Gaussian fitting of their point-spread function with a mean localization accuracy of 32±8 nm (Fig. 2a,e and Supplementary Fig. 3). Worms were additionally imaged by SM CALM-spFRET after microinjection of M3-A647 peptides. At the sarcolemma, individual complemented CD4-split-GFPs were identified as diffraction-limited and colocalized diffusing spots that emitted in both GFP and A647 detection channels when excited at 488 nm (Fig. 2c). Single bleaching steps and blinking events where both GFP and A647 fluorescence signals disappeared (Fig. 2c) indicated an effective intramolecular spFRET between CD4-split-GFP and M3-A647 and confirmed our ability to specifically target, activate, detect and track individual transmembrane CD4-split-GFP directly in *C. elegans*.

When analysing the diffusion of activated CD4-split-GFP (6,193 trajectories, 21 muscle cells, five worms) by probability distribution of the squared displacements (PDSD)^17^, three diffusion regimes were identified (Fig. 2d). A first high-mobility regime (10%) with a diffusion coefficient *D*_CD4−1_=1.14 × 10^−1^±5 × 10^−3 ^μm^2 ^s^−1^ showed free diffusion for a length scale of up to 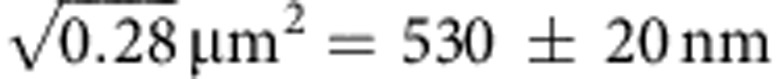 (Fig. 2f). Two additional low-mobility regimes with diffusion coefficients *D*_CD4−2_=2.34 × 10^−2^±3.4 × 10^−3 ^μm^2 ^s^−1^ (38%) and *D*_CD4−3_=3.31 × 10^−3^±8.0 × 10^−4 ^μm^2 ^s^−1^ (52%) were each confined to membrane microdomains 420±20 and 110±4 nm in diameter, respectively (Fig. 2f). The diffusion coefficients and confinement sizes of these three regimes are consistent with the generally heterogeneous and restricted diffusion of other transmembrane proteins in cultured muscle cells^18^,^19^; however, the largely confined and slow diffusive behaviours of CD4-split-GFP *in vivo* likely stem from extracellular matrix effects that are absent in cultured cells and from tracking at lower temperatures. Importantly, these results demonstrate that the nanoscale diffusion of individual membrane proteins and their interactions with plasma membrane microdomains can be quantitatively studied at the SM level in intact animals using CALM.

### CALM imaging of plasma membrane L-type VDCC in *C. elegans*

**Figure 3:**
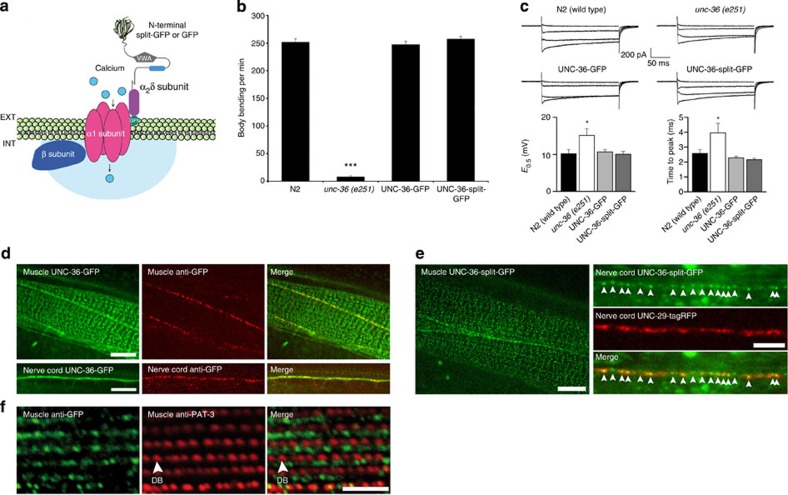
Imaging of plasma membrane L-type VDCCs in live *C. elegans* by genetic engineering of UNC-36 fusion proteins. (**a**) Schematic of split-GFP or full GFP fusion to UNC-36 (α_2_δ subunit) and its association with the α_1_ and β subunits to form active transmembrane VDCC. (**b**) *C. elegans* thrashing assays showing impaired motility in *unc-36*(*e251*) null allele mutant worms and functional rescue upon UNC-36-GFP and UNC-36-split-GFP gene insertion. The mean number of body bending per min (±s.d.) was determined for *n*=20 worms for all conditions (Welch‘s *t*-test, ***P<0.001). (**c**) Representative traces of inward VDCC currents recorded from body muscle cells in response to depolarizing pulses from −60 mV to −20, 0, +20 and +40 mV. Compared with wild-type N2 worms (*n*=8), the increase in activation midpoint potential (E_0.5_±s.e.m.) and time-to-peak kinetics of inward currents (±s.e.m.) observed for *unc-36* (*e251*) worms (*n*=7) are restored to wild-type levels in UNC-36-GFP (*n*=7) and UNC-36-split-GFP worms (*n*=8; *E_0.5_: ANOVA test *P*=0.0141, Dunnett’s post test *P*<0.05 for *unc-36(e251)*, *P*>0.05 for others; *time-to-peak: Kruskal–Wallis test *P*=0.0026, Dunn’s post test *P*<0.05 for *unc-36(e251)*, *P*>0.05 for others). (**d**) Ensemble confocal fluorescence imaging of UNC-36-GFP and alexa-594 anti-GFP antibody in live *C. elegans*. UNC-36-GFP is expressed in muscle cells and motor neurons at the nerve cords. Anti-GFP antibodies target only the edge of muscle cells and the nerve cords. Scale bars: 5 μm. (**e**) Ensemble dCALM confocal imaging of UNC-36-split-GFP in live *C. elegans* expressing the acetylcholine receptor subunit UNC-29-tagRFP. Membrane UNC-36-split-GFP is detected at the sarcolemma of muscle cell bodies (left) and within neuromuscular synapses (red) along the nerve cords (arrowheads, right). Scale bars: 5 μm. (**f**) Immunofluorescence detection of GFP and of the beta-1 integrin PAT-3 showing UNC-36-split-GFP localized in the A-band region of sarcomeres between rows of dense bodies (DBs). Scale bar: 5 μm.

To image VDCC by CALM, we fused split-GFP to the N terminus of the α_2_δ VDCC subunit (UNC-36) that associates with both neuronal (UNC-2)^20^ and muscular (EGL-19)^21^ α_1_ subunits to form active membrane Ca^2+^ channels (Fig. 3a). *unc-36* null allele (*e251*) worms were rescued by reintroducing UNC-36-split-GFP under the control of the endogenous *unc-36* promoter via single copy insertion using *Mos*SCI protocols^22^. Mutant worms were also rescued with UNC-36-GFP to compare CALM imaging with conventional GFP imaging of UNC-36. The resulting integrated worms had fully rescued VDCC activities. The phenotypic impairment of locomotion for UNC-36 mutation was completely restored in thrashing assays and the conducting activities of VDCC were identical to that of wild-type channels (Fig. 3b,c and Supplementary Fig. 4).

In live worms, UNC-36-GFP localized in muscle cells and motor neurons. Muscular UNC-36-GFPs were distributed at the boundary of body-wall muscles and as a punctuated pattern at the sarcolemma, while neuronal UNC-36-GFPs were distributed at the nerve cords (Fig. 3d). To assess the effective plasma membrane translocation of UNC-36 in these tissues, live worms were microinjected with fluorescently labelled anti-GFP antibodies. Anti-GFP staining was observed at the boundary of body-wall muscle cells and as punctuates along the nerve cords but not at the sarcolemma (Fig. 3d). The lack of sarcolemma staining was likely because of the inability of large antibodies to access crowded extracellular spaces at the interface between the epidermal and body-wall muscle cells. Indeed, when we injected much smaller M3 peptides and performed dCALM imaging, GFP fluorescence from membrane-activated UNC-36-split-GFP was specifically detected at the nerve cords and the muscle sarcolemma (Fig. 3e). Activated GFP signals were weaker than those of UNC-36-GFP suggesting a low, yet systematic, plasma membrane translocation of muscular and neuronal UNC-36. Activated UNC-36-split-GFP was localized at the membrane of neuromuscular synapses based on colocalization with the fluorescent acetylcholine receptor subunit UNC-29-tagRFP (Fig. 3e). This position is consistent with the expected location of VDCC in presynaptic active zones of motor neurons^20^. UNC-36-split-GFP was also detected at the sarcolemma, consistently with its interaction with the muscular EGL-19 α_1_ subunit of VDCC^21^. It localized in the A-band region, between rows of dense bodies, the structures that anchor sarcomeric actin filaments to the sarcolemma (Fig. 3f).

These results demonstrate that CALM affords specific *in vivo* targeting and imaging of membrane Ca^2+^ channels within confined extracellular spaces at the basal lamina–muscle interface and at neuromuscular synapses in *C. elegans* tissues.

### Tracking of individual VDCC using CALM in *C. elegans*

**Figure 4:**
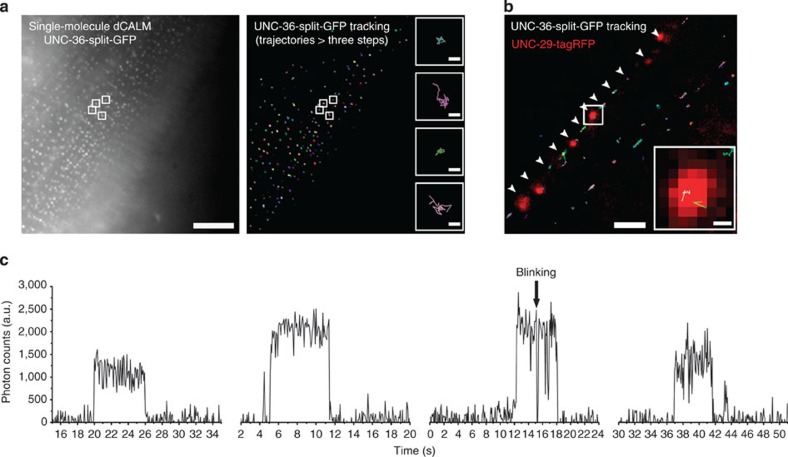
Single-molecule dCALM imaging and tracking of individual muscular and neuronal VDCCs in *C. elegans*. (**a**) Sum intensity image of a single-molecule dCALM movie (left) and corresponding trajectory map of individual VDCC (right) on a body-wall muscle cell. Trajectories with at least three steps are shown. Scale bar: 10 μm. Inset: representative diffusion trajectories of single VDCC from the sum and tracking images (white squares). Scale bar: 100 nm. (**b**) Overlay image of individual VDCC trajectories with UNC-29-tagRFP-labelled motor neuron synapses (red). Individual channels diffuse at the nerve cord (arrowheads) within synapses (inset). Scale bars: 2 μm and 200 nm (inset). (**c**) Background-corrected fluorescence intensity time traces of individual muscular or neuronal VDCC.

We then used SM dCALM and HILO excitation to track individual VDCC in live worms. At the sarcolemma, activated VDCC appeared as individual diffraction-limited fluorescent spots during imaging (Supplementary Movie 3) and were distributed as punctuates on muscle cells (Fig. 4a), in a pattern similar to that observed using confocal microscopy. For each muscle cell, hundreds of individual VDCC could be tracked with a precision better than 30 nm (Supplementary Fig. 3). Muscle-wide trajectory maps revealed a largely confined diffusion of the channels at the sarcolemma (Fig. 4a). Individual VDCC tracked along the nerve cords were also seen diffusing within neuromuscular synapses when we overlapped UNC-36-split-GFP trajectory maps with UNC-29-tagRFP images for worms co-expressing both fusion proteins (Fig. 4b). The single emitter nature of both muscular and neuronal VDCC was confirmed using fluorescence intensity time trace analyses that displayed sudden activation, single-step photobleaching and blinking events typical of SM detection by dCALM^12^ (Fig. 4c). No activation of UNC-36-split-GFP was observed when imaging non-injected integrated worms (Supplementary Movie 4).

### Muscle contraction modulates VDCC diffusion in nanodomains

Taking advantage of the nanometre-precision imaging capabilities of dCALM, we compared the nanoscale distribution and diffusion of individual VDCC in resting body-wall muscles of worms immobilized with polystyrene beads and in muscles undergoing sustained contraction for worms anaesthetized with the cholinergic agonist levamisole.

**Figure 5:**
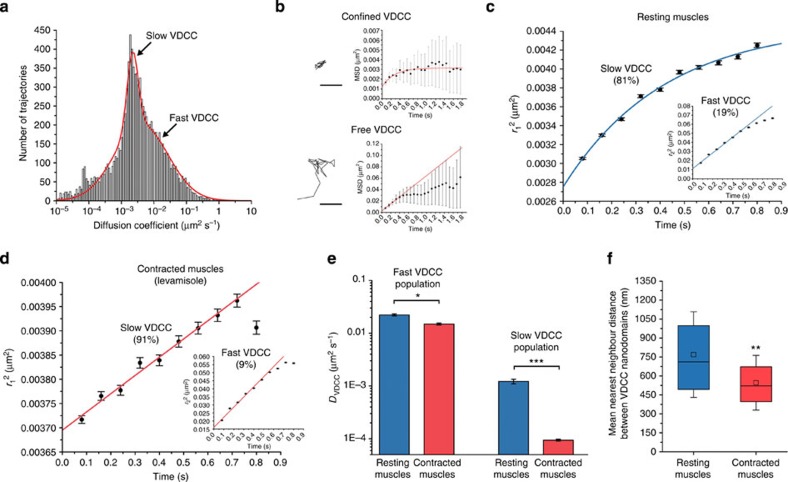
Diffusion analysis of individual VDCC at the sarcolemma of normal *C. elegans* worms expressing UNC-36-split-GFP. (**a**) Distribution of diffusion coefficients for 10,481 VDCC trajectories (39 muscle cells, six worms) determined from individual MSD analysis and showing that two populations of slow and fast diffusing channels co-exist at the sarcolemma of resting muscles. (**b**) Example of individual VDCC trajectories and the evolution of their MSD over time. VDCC undergo either confined or short-scale free diffusion as determined using 2D diffusion models that best fit the MSD (red). Scale bars: 200 nm. (**c**) Ensemble probability distribution of the squared displacement (PDSD) analysis of VDCC diffusive behaviours (10,481 VDCC, 39 muscle cells, six worms) in resting muscles. (**d**) Ensemble PDSD analysis of VDCC diffusive behaviours (36,325 VDCC, 62 muscle cells, 18 worms) in muscles under sustained contraction with levamisole. (**e**) Diffusion coefficients (±s.d.) for both fast and slow VDCC populations in resting and contracted muscles (F-test, *P<0.05, ***P<0.001). (**f**) VDCC nanodomain nearest-neighbour distances at the sarcolemma of resting and contracted muscles. The central squares and bars represent the mean of the distribution and its median, respectively. The box length represents the interquartile range and the error bars the s.d. of the mean (Wilcoxon sign-rank test, **P<0.01 compared with resting muscles).

In resting muscles, two populations of slow and fast diffusing VDCC undergoing confined or short-scale free diffusion were identified at the sarcolemma (10,481 trajectories, 39 muscle cells, six worms, Fig. 5a,b). Ensemble PDSD analyses revealed that most channels (81%) diffused slowly (*D*_vdcc−slow_=1.22 × 10^−3^±1.2 × 10^−4 ^μm^2 ^s^−1^) in membrane nanodomains 82±2 nm in diameter (Fig. 5c,e and Supplementary Table 1). The second VDCC population (19%) diffused 20-fold faster (*D*_vdcc−fast_=2.22 × 10^−2^±1 × 10^−3 ^μm^2 ^s^−1^) and underwent free diffusion for a length scale of 230±8 nm (Fig. 5c,e and Supplementary Table 1), after which membrane obstacles induced a subdiffusive behaviour. Both populations were independently distributed at the sarcolemma (Supplementary Fig. 5), did not change diffusion and diffused more slowly than CD4-split-GFP, indicating that we imaged two behaviours of stably assembled VDCC rather than a dynamic association of the glycosylphosphatidylinositol-anchored UNC-36 (ref. 23) with the channels’ α_1_ subunit.

Additional Ripley’s K function and nearest-neighbour distance analyses of the clustering and dispersion pattern of VDCC nanodomains at the sarcolemma indicated that they were not clustered but significantly more dispersed than expected for complete spatial randomness, with a mean nearest-neighbour distance of 769±339 nm (Fig. 5f and Supplementary Fig. 6). This distance, below the 1-μm average width of sarcomeres in adult *C. elegans*^24^ and above the width of one-half A-band (~350 nm), suggests that individual sarcomere contains two VDCC nanodomains. Because of the stochastic nature of CALM, we could not accurately determine the exact number of VDCC per nanodomain. However, when we injected worms with high concentrations of M3 peptides to activate as many VDCCs as possible at the sarcolemma, a count of single photobleaching steps in individual nanodomains and additional quantitative analyses of their fluorescence intensity distribution indicated that some nanodomains comprise at least 10 VDCC (Supplementary Fig. 7).

In muscles undergoing sustained contraction induced by levamisol (36,325 trajectories, 62 muscle cells, 18 worms), slow diffusing VDCC (91%) were nearly immobile with a 13-fold decrease in lateral diffusion coefficient compared with resting muscles (*D*_Leva vdcc−slow_=9.4 × 10^−5^±4 × 10^−6 ^μm^2 ^s^−1^, *P*<0.001, Fig. 5d,e). No confinement was detected, indicative of a strong reduction of the nanodomains’ size or the near-immobilization of VDCC within intact nanodomains. The diffusion of the fast VDCC population (9%) was moderately reduced by 33% compared with resting muscles (*D*_Leva vdcc−fast_=1.49 × 10^−2^±7 × 10^−4 ^μm^2 ^s^−1^, *P*<0.05); however, the channels’ free diffusion distance was unchanged at 224±9 nm (Fig. 5d,e and Supplementary Table 1). Interestingly, the nearest-neighbour distance between nanodomains significantly decreased to 546±217 nm compared with resting muscles (*P*<0.01, Fig. 5f and Supplementary Fig. 6). Transient reductions in nanodomain interdistance were also observed during sporadic muscle contractions of immobilized worms (Supplementary Movie 5) suggesting that, under sustained or active contractions, the sarcolemma undergoes a deformation that brings stable VDCC nanodomains closer to each other in a process reminiscent of sarcolemma festooning: a ballooning of the membrane between dense bodies when muscles are shortened to less than their resting length^25^.

Thus, the nanoscale mobility and the distribution of VDCC are modulated by the tonic state of muscles. Our observations that a large majority of VDCC are constrained in two nanodomains on each sarcomere indicate that VDCC action potentials and extracellular Ca^2+^ influx are spatially controlled in *C. elegans* body-wall muscles and that they are further regulated, on the nanometre scale, by a near-immobilization of the channels during sustained cholinergic contractions. The nanoscale confinement of most VDCCs and the effect of muscle contraction on their diffusion suggest that this dominant population of Ca^2+^ channels is primarily involved in electromechanical coupling at the sarcolemma.

### Dystrophin impacts the nanoscale diffusion of sarcolemma VDCC

**Figure 6:**
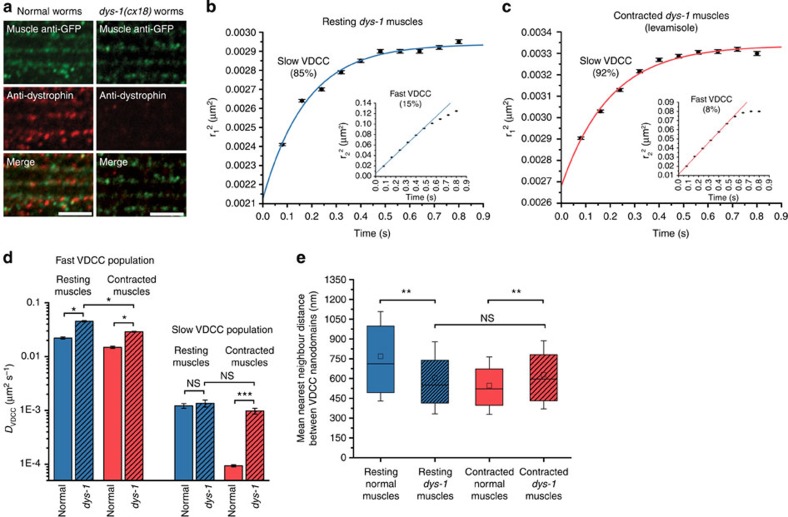
Diffusion studies of individual VDCC at the sarcolemma of *dys-1*(*cx18*) mutants expressing UNC-36-split-GFP. (**a**) Immunofluorescence staining of GFP and dystrophin in normal and *dys-1*(*cx18*) worms expressing UNC-36-split-GFP. Scale bars: 5 μm. (**b**) Ensemble PDSD analysis of VDCC diffusive behaviours (17,273 VDCC, 32 muscle cells, five worms) in resting *dys-1* mutant muscles. (**c**) Ensemble PDSD analysis of VDCC diffusive behaviours (40,068 VDCC, 33 muscle cells, six worms) in contracted *dys-1* mutant muscles with levamisole. (**d**) Diffusion coefficients (±s.d.) for both VDCC populations in resting and contracted muscles of normal and *dys-1* mutant worms (F-test, *P<0.05, ***P<0.001, NS: nonsignificant). (**e**) VDCC nanodomain nearest-neighbour distances at the sarcolemma of resting and contracted muscles for normal and *dys-1* mutant worms. Error bars represent the s.d. of the mean (Wilcoxon sign-rank test, **P<0.01, NS: nonsignificant).

At the sarcolemma, the interaction of dystrophin with phospholipids, γ-actin and the dystrophin-glycoprotein complex^26^,^27^ could favour its role as a tension-sensing scaffold and a load-bearing apparatus capable of transducing muscle tension to membrane-embedded ion channels^28^,^29^. To investigate the role of dystrophin as a potential modulator of VDCC lateral mobility, we imaged dystrophin-deficient *C. elegans dys-1*(*cx18*) mutant worms expressing UNC-36-split-GFP (Fig. 6a) by SM dCALM.

At the macroscale there was no obvious difference in the sarcolemma distribution of VDCC between *dys-1* mutant and normal worms (Supplementary Movie 6). However, clear changes in sarcolemma scaffolding and fluidity were observed when we tracked individual channels. In resting *dys-1* mutant muscles (17,273 trajectories, 32 muscle cells, five worms), the diffusion coefficient of slow VDCC (85%) was not significantly different from that of normal worms (*D*_*dys-1* VDCC−slow_=1.36 × 10^−3^±2.0 × 10^−4 ^μm^2 ^s^−1^, *P*=0.12, Fig. 6b,d); however, their confinement size was reduced to 58±2 nm in diameter (Supplementary Table 1), indicating that dystrophin is required to maintain the confining area of VDCC nanodomains but not their fluidity. The fast VDCC population (15%) diffused twofold faster than in normal worms (*D*_*dys-1* VDCC−fast_=4.54 × 10^−2^±1.2 × 10^−3 ^μm^2 ^s^−1^, *P*<0.05, Fig. 6b,d), an increased lateral mobility similar to that observed for other membrane proteins in muscle cells lacking dystrophin^30^. As dystrophin stiffens membranes that mimic the sarcolemma^31^, we attribute this apparent higher sarcolemma fluidity to a loss of membrane stiffness in dystrophin-mutant muscles. The free diffusion distance of fast VDCC was increased to 303±10 nm compared with resting normal muscles (Supplementary Table 1), suggesting that dystrophin is also involved in maintaining sarcolemma scaffolds outside VDCC nanodomains in body-wall muscles. VDCC nanodomains remained well dispersed but with a nearest-neighbour distance reduced to 606±273 nm compared with normal worms (*P*<0.01, Fig. 6e and Supplementary Fig. 6). This enhanced festooning of the sarcolemma and its reduced stiffness are both consistent with dystrophin being a load-bearing element capable of minimizing membrane strain by damping transverse cytoplasmic forces that push towards the sarcolemma under resting muscular tension. The smaller confinement area but unchanged diffusion coefficient of nanodomain-associated VDCC further suggests that, in normal resting *C. elegans* muscles, dystrophin does not bind directly to the channels but participates in modulating the nanodomain confinement size and the nanoscale spatial distribution of VDCC.

In *dys-1* mutant worms anaesthetized with levamisole (40,068 trajectories, 33 muscle cells, six worms), the slow VDCC population (92%), which was nearly immobile in normal contracted muscles, displayed diffusive behaviours similar to that observed in resting muscles lacking dystrophin, with a confined diffusion within nanodomains 52±2 nm in diameter (*D*_Leva*dys-1*VDCC−slow_=9.8 × 10^−4^±1.2 × 10^−4 ^μm^2 ^s^−1^, *P*=0.06,Fig. 6c,d and Supplementary Table 1). The fast VDCC population (8%) was less mobile by 35% compared with resting *dys-1* mutant muscles (*D*_Leva *dys-1* VDCC−fast_=2.90 × 10^−2^±5 × 10^−4 ^μm^2 ^s^−1^, *P*<0.05) and diffused freely over a nonsignificantly changed length scale of 281±6 nm (Fig. 6c,d and Supplementary Table 1). The nearest-neighbour distance between VDCC nanodomains was similar to that of resting dystrophin-mutant muscles at 628±257 nm (Fig. 6e and Supplementary Fig. 6). This apparent lack of additional sarcolemma festooning and the unchanged diffusive behaviour of nanodomain-associated VDCC in *dys-1* mutant muscles under sustained cholinergic stimulation indicate that, in *C. elegans*, a functional dystrophin is required for an effective modulation of VDCC nanoscale dynamics at the sarcolemma during contractions.

Thus, dystrophin is a key scaffolding protein of the sarcolemma where it plays a dual role as a load-bearing apparatus and a tension transducer that has an impact on the nanoscale diffusion of membrane-embedded VDCC in response to changes in muscle tones. By showing that dystrophin is involved in modulating the confinement size of VDCC that normally generate action potentials and extracellular Ca^2+^ influx in muscle cells, our SM dCALM data suggest that it participates, directly or indirectly, in electromechanical coupling at the membrane of body-wall muscles in live *C. elegans*.

## Discussion

Using far-field fluorescence microscopy and CALM, we achieved highly specific, SM sensitivity and nanometre accuracy imaging of membrane proteins within confined tissue regions in intact living *C. elegans* worms. We showed that microinjected CALM complementary peptides diffusing throughout the body of animals can activate fluorescent signals in engineered muscular and neuronal tissues by targeting and binding irreversibly to cell surface split-GFP fusion proteins. By tuning the concentration of injected peptides, ensemble or continuous SM CALM imaging could be performed by direct detection of activated split-GFP or by spFRET with targeted near-infrared fluorophores regardless of protein expression levels and of peptide nonspecific binding. Compared with SM imaging with conventional FP^6^, CALM provides low-background and membrane-specific SM detection without a need for photobleaching or alteration of protein expression and thus permits SM studies at physiologically relevant expression levels directly in live animals. While we primarly focused on membrane protein imaging, similar SM CALM detection of intracellular biomolecules may be achieved by co-expressing split-FP fusions with genetically encoded M3 peptide sequences. Chemically or light-inducible transcriptional elements might be used to control the expression of complementary peptides and maintain an SM detection regime. In principle, multiplex *in vivo* CALM imaging can also be realized with additional split-CFP and split-YFP fusions and by varying the fluorophores employed for CALM-spFRET.

In *C. elegans*, we tracked thousands of individual transmembrane proteins and channels with subresolution precision and quantitatively studied their diffusion in membrane micro- and nanodomains directly in live adult animals. In contrast to ensemble and diffusion-limited measurements by fluorescence recovery after photobleaching or FCS, single-particle tracking by CALM provides detailed analyses of protein locations and subpopulation dynamics at the nanoscale even for slow diffusing and confined membrane biomolecules. While our technique can be implemented in any animal species amenable to fluorescence imaging, *C. elegans* provides many advantages for *in vivo* SM studies. Its optical transparency, simple anatomy, small size and easy propagation facilitate sample preparation and peptide injection for SM CALM. These advantages, combined with the means to generate knock-in strains^32^,^33^ and the availability of large bioresources of *C. elegans* mutants open the possibilities of correlating the nanoscale behaviour of individual biomolecules imaged by CALM with phenotypic changes occurring at various developmental or disease stages, as illustrated by our study of VDCC dynamics in normal and dystrophin-mutant adult worms.

In this study, we reveal that dystrophin is a load-bearing scafolding protein of *C. elegans* body-wall muscles that not only influences membrane stiffness but also tranduces muscle tension to membrane-embeded VDCC. We show that, in response to changing muscle tones, dystrophin participates in modulating the nanoscale diffusion of two sarcolemmal populations of VDCC: a majority VDCC fraction undergoing slow and confined diffusion in ordered membrane nanodomains, and a minority fraction of channels diffusing faster and more randomly in other parts of the sarcolemma. As observed for comparable Ca^2+^ channels in cardiac myocytes^34^, the different diffusion characteristics and locations of these two VDCC populations suggest that they might play distinct roles for extracellular Ca^2+^ signalling on body-wall muscles. The fact that muscle contractions and the lack of functional dystrophin primarily influence nanodomain-associated VDCC implies that this subpopulation of Ca^2+^ channels and dystrophin itself are likely involved in electromechanical coupling in *C. elegans*, a process that requires precise spatial and temporal controls of Ca^2+^ influx in animal muscular tissues. The smaller, faster and more randomly distributed diffusing population of VDCC might regulate other Ca^2+^-dependent cellular processes in muscle cells.

The exact nature of VDCC nanodomains remains to be determined. While caveolae have been proposed to be sites of VDCC confinement in mammalian muscle cells^35^,^36^, there is as yet no evidence of caveolae formation at the sarcolemma of *C. elegans*, despite the expression of two caveolin isoforms^37^,^38^. Rather than caveolae, flat caveolin scaffolds^39^ or membrane rafts^40^ are potential nanodomains that could confine VDCC and induce their slow diffusion. Many *C. elegans* glycosylphosphatidylinositol-anchored proteins, such as UNC-36, co-fraction with caveolin in detergent-resistant membranes^41^ and in mammalian cells, α_2_δ subunits partition with other VDCC subunits in lipid rafts^42^. In cells, these membrane domains can slow down the lateral diffusion of proteins by a 10- to 20-fold ratio^18^,^43^. Our observations that the diffusion of VDCC inside or outside nanodomains differ by a comparable ratio suggest that they might be confined by similar raft-like domains at the sarcolemma.

We speculate that the confined diffusion of VDCC in sarcolemmal nanodomains is important for normal muscle functions in *C. elegans*. The confinement of VDCC and their near-immobilization during contractions in normal body-wall muscles might amplify small extracellular Ca^2+^ flux from action potentials into high local Ca^2+^ concentrations by nanodomain Ca^2+^ boosting^44^. This nanometre-scale localization of VDCC Ca^2+^ loads could reduce cross-talks between intracellular Ca^2+^ sources and increase the opening probability of Ca^2+^-dependent ryanodine/UNC-68 receptors in the closely apposed sarcoplasmic reticulum^45^ to potentiate muscle contractions^46^,^47^. The impaired modulation of VDCC confinement observed here in dystrophin-mutant muscles might additionally have an impact on nanodomain coupling between VDCC and SLO-1 current rectifying BK channels^48^,^49^ which, when defective, increases the excitability of muscle cells^15^ and induces the hyper-contracted phenotype of *dys-1*(*cx18*) worms^50^. Overall, it appears that dystrophin plays a key role for the nanoscale modulation of VDCC dynamics at the sarcolemma in *C. elegans* body-wall muscle cells. Such a function was not anticipated based on conventional imaging but might participate to the impairment of Ca^2+^ homeostasis and of normal muscle function in Duchenne muscular dystrophy^51^,^52^.

In conclusion, SM CALM imaging opens new avenues to explore the basic principles of homeostatic controls and the molecular basis of diseases at the nanometre scale in intact animal models by providing subresolution localization of individual membrane proteins and unprecedented insights into their diffusive behaviours within live tissues.

## Methods

### *C. elegans* strains

*C. elegans* strains were maintained on NGM agar plates at 20 °C on *Escherichia coli* OP50 lawns. N2 Bristol wild-type worms, CD4-split-GFP (*kyEx1940[Pmyo-3::SL2::GFP1-10-CD4; Podr-1::SL2::DsRed]*) worms a kind gift of Dr Cornelia Bargmann, strain EG4322 (*ttTi5605*II; *unc-119*(*ed9*)III) and *unc-36*(*e251*) worms from the Caenorhabditis Genetics Center, *dys-1*(*cx18*) worms a kind gift from Dr Kathrin Gieseler, UNC-36-GFP (*krSi3[UNC-36::GFP]*; *e251)* worms, UNC-36-split-GFP (*krSi6[UNC-36::GFP 1-10]; e251*) worms, UNC-36-split-GFP dystrophin-mutant (*krSi6[UNC-36::GFP 1-10]; e251; cx18*) worms and UNC-36-split-GFP/UNC-29-tagRFP (*krSi6[UNC-36::GFP 1-10]; kr208(unc-29::tagRFP); e251*) worms were used in this study.

### Plasmid construction and *C. elegans* genetics

UNC-36-split-GFP was obtained by NheI and SacI insertion of the split-GFP-coding sequence (GFP 1–10) after the signal peptide sequence in *unc-36* cDNA^20^ by PCR fusion. A synthetic *C. elegans* intron was also introduced upstream of GFP 1–10 to improve transgene expression. This transgene sequence was inserted in a pCFJ151 vector backbone (Addgene) to create the targeting vector pHZ043 and generate integrated animals by direct insertion on chromosome II using *Mos*SCI protocols^22^. An EGFP sequence was fused to UNC-36 cDNA using the same approach, resulting in targeting vector pHZ025.

For *Mos*SCI experiments, a mixture of plasmids including the targeting vectors pHZ043 or pHZ025 at 50 ng μl^−1^, the *Mos* transposase expression vector pJL43.1 at 50 ng μl^−1^ and the transformation markers *Pmyo-3::GFP* and *Prab-3::GFP* each at 10 ng μl^−1^ were co-injected in worm strain EG4322. *Mos*SCI insertion progenies were screened by restoration of locomotion phenotypes and absence of transformation markers. *Mos*SCI insertion was verified using PCR. *Mos*SCI worms were then crossed with *unc-36* null allele (*e251*) mutant worms to generate UNC-36-split-GFP and UNC-36-GFP worms. UNC-36-split-GFP worms were further crossed with a worm strain expressing the postsynaptic acetylcholine receptor fused to a red fluorescent protein (UNC-29-tagRFP) to generate worms dually expressing UNC-36-split-GFP/UNC-29-tagRFP. UNC-36-split-GFP worms were also crossed with dystrophin-deficient *dys-1*(*cx18*) mutant worms to generate dystrophin-mutant worms expressing UNC-36-split-GFP. All crosses were verified using PCR.

### *C. elegans* phenotyping

Effective rescue of VDCC activity by integrated expression of UNC-36-GFP and UNC-36-split-GFP was verified in thrashing assays where 20 adult worms were transferred separately in a drop of M9 medium at 20 °C and thrashes were counted in 1-min intervals after 2 min recovery. Data are reported as the mean number of body bends per min±s.d. of the mean. Statistically significant differences are estimated by a Welch two sample *t*-test.

### Electrophysiology

Following microdissection of *C. elegans*, electrophysiological recordings of membrane currents and potentials were performed in the whole-cell configuration using an RK-400 patch-clamp amplifier (Bio-Logic)^53^. Acquisition and command voltage were controlled using the pClamp9 software driving a 1322A Digidata (Molecular Devices). Data were analysed and graphed using Origin software (OriginLab). The resistance of recording pipettes ranged between 2.5 and 2.8 MΩ. Recordings were performed after 1 min dialysis only on cells exhibiting resistances above 800 MΩ. Capacitance and resistance were not compensated.

The bath solution contained 140 mM Tetraethylammonium chloride (TEACl), 6 mM CaCl_2_, 5 mM MgCl_2_, 3 mM 4-Aminopyridine (4-AP), 10 mM Hepes and sucrose at 337 mosm l^−1^ (pH 7.2). The pipette solution contained 140 mM CsCl, 5 mM TEACl, 5 mM EGTA, 5 mM Hepes, 4 mM MgATP and sucrose at 328 mosm l^−1^ (pH 7.2). The membrane potential was hold at −60 mV, and currents were recorded by applying 200-ms voltage steps from −70 to +70 mV with 10-mV increments. Leak currents were subtracted from all recordings. Current–voltage relationships for N2 wild-type (*n*=8), *unc-36(e251)* (*n*=7), UNC-36-GFP (*n*=7) and UNC-36-split-GFP (*n*=8) worms were established by measuring the currents at the peak of the current and were fitted with the following equation:


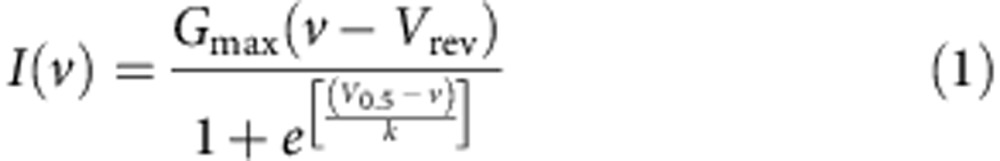


where *I*(v) is the measured density of current, *v* the test voltage pulse, *G*_max_ the maximum conductance, *V*_rev_ the reversal potential, *V*_0.5_ the half-activation voltage and *k* the steepness factor. Potentials of half-activation, *E*_0.5_ were obtained by fitting, for each muscle cell, the current–voltage relationships of the inward currents measured at the peak of the currents by equation (1). Time-to-peak kinetic was measured for the maximal current traces (+20 mV). The mean *E*_0.5_ and the mean time-to-peak values are reported with error bars representing the s.e.m.

All chemicals were obtained from Sigma-Aldrich and all experiments were performed at room temperature.

### Immunohistochemistry

Mix-stage worms were freeze-fractured and fixed in methanol/acetone according to freeze fracture protocols^54^; however, only adult worms were imaged. For immunodetection of split-GFP fusion proteins, primary rabbit anti-GFP antibodies (Invitrogen A11122, dilution 1/500) and secondary polyclonal goat anti-rabbit Alexa-488-conjugated antibodies (Invitrogen A11008, dilution 1/800) were used. For immunostaining of dense bodies, mouse MH25 antibodies against PAT-3 β-integrin^55^ (dilution 1/200) and secondary polyclonal goat anti-mouse antibodies conjugated to Cy3 (Invitrogen A10521, dilution 1/500) were used. For immunostaining of dystrophin, mouse monoclonal antibodies against *C. elegans* dystrophin (BioTem 1H7-1B3-2C7-3F5, dilution 1/200), a kind gift from Dr Kathrin Gieseler, and secondary polyclonal goat anti-mouse antibodies conjugated to Cy3 (dilution 1/500) were used.

### Worm microinjection

Worms were placed on dried 2% agarose injection pads in a drop of mineral oil (Sigma, H8898) and were forced to adhere to the pads by gentle brushing of the animal body. For ensemble dCALM and CALM-spFRET imaging, purified synthetic complementary M3-biotin and M3-A647 peptides^12^ were diluted in PBS at concentrations of 5 mM and 200 μM, respectively, before being microinjected into the worms pseudoceolomic cavity in the head region of the animals using a microinjector (Femtojet, Eppendorf) and FemtotipII microinjection capillaries. Worms were microinjected in three to five steps of 0.3 s with a few hundreds of femtolitres of the peptide solutions. Worms were allowed to recover in an M9 drop on NGM plates at 15 °C for 1 h and were then transferred to 20 °C for at least 3 h before imaging. For SM dCALM and SM CALM-spFRET of CD4-split-GFP, microinjections were performed with M3-biotin and M3-A647 peptide concentrations reduced to 200 and 20 μM, respectively, and worms’ recovery times were limited to 30–60 min before imaging. For SM dCALM of VDCC, worms were injected with higher M3-biotin concentrations than for SM dCALM of CD4-split-GFP and longer recovery times were employed to compensate for the low endogenous membrane expression of UNC-36-split-GFP. M3-biotin peptides at 1 mM were microinjected and worms were allowed to recover for 3–12 h at 15 °C before imaging. For *in vivo* membrane staining of UNC-36-GFP, anti-GFP Alexa-594 antibodies (Invitrogen A-21312) were diluted 200-fold in injection buffer (20 mM K_2_HPO_4_, 3 mM K^+^ citrate, 2% PEG 3000, pH=7.4) and were injected into the pseudocoelomic cavity^56^ in the head region before recovery in an M9 drop on a NGM plate at 20 °C for 3–6 h and imaging.

### Ensemble microscopy imaging

Ensemble GFP, dCALM and CALM-spFRET imaging of live worms were performed on a confocal Leica TCS SP5AOBS microscope or a Leica DM5000B microscope equipped with a CSU10 spinning disc confocal scanner system (Yokogawa). Appropriate laser excitation wavelengths and emission filtering were used to image GFP and activated split-GFP fusions (488 nm excitation), anti-GFP antibodies or UNC-29-tagRFP (561 nm excitation) and M3-A647 peptides (633 nm excitation). FRET from complemented CD4-split-GFP to M3-A647 was detected by single laser excitation at 488 nm and simultaneous detection in the GFP and the A647 emission channels. Live worms were anaesthetized with 0.2% sodium azide or 50 mM muscimol before being mounted between 2% agarose pads and microscope coverslips for imaging. Freeze-fractured and immunostained worms were imaged on the same microscopes, except for immunostained CD4-split-GFP worms that were imaged by wide-field epifluorescence.

### Single-molecule microscopy imaging

SM dCALM and SM CALM-spFRET of CD4-split-GFP in live worms were performed on an inverted IX70 Olympus microscope equipped with custom-built total internal reflection (TIR) and highly incline and laminated optical (HILO) elements, a × 100 1.45 NA objective, a DV2 dual-view system (Photometrics), a QuantEM:512SC EMCCD (Photometrics) and appropriated filters^12^. SM dCALM imaging of VDCC and simultaneous dual-colour imaging of UNC-29-tagRFP was performed on an inverted Nikon Eclipse Ti-E microscope equipped with TIR and HILO optics, 488, 561 and 647 nm fibre-coupled excitation lasers, a × 100 1.49 NA objective, a two-camera imaging splitter (Andor) and two iXon EMCCD cameras (Andor). A multiband pass ZET405/488/561/647x excitation filter (Chroma), a quad-band ZT 405/488/561/647 dichroic mirror (Chroma), an emission splitting FF560-FDi01 dichroic mirror (Semrock) and two emission filters at 525/50 nm (Semrock) and 600/50 nm (Chroma) for GFP and tagRFP, respectively, were used.

HILO imaging of worms was achieved by moving circularly polarized laser excitation beams away from the optical axis at the back-focal plan of the objectives and at a position slightly off from the location normally required to obtain a critical TIR angle. For both optical set-ups, the size of pixels in images was determined by imaging a micrometre reticle and the alignment of emission channels was performed by imaging 40-nm diameter TransFluoSphere beads (488/685 nm, Invitrogen).

Non-anaesthetized worms (resting muscles) were deposited on 2% agarose pads in a drop of M9 containing 0.1 μm polystyrene beads at 2.5% per volume (Polyscience) and were mounted with a microscope coverslip^57^. Tracking was only performed in-between sporadic muscle contractions. Worms anaesthetized with levamisole (contracted muscles) were first deposited in a drop of 1 mM levamisole (Sigma) for 10–15 min before being mounted in levamisole between 2% agarose pads and coverslips. In each worm, multiple muscle cells were imaged at room temperature by continuous laser excitation and with camera acquisition rates at 100 ms per frame for CD4-split-GFP and 80 ms per frame for VDCC.

### Tracking and diffusion analyses

SM tracking and diffusion analyses were performed using SlimFast, a single-particle detection and tracking software written in Matlab and based on multiple-target tracing algorithms^58^, which was generously provided by Christian Ritcher and Jacob Piehler. We used parallel Matlab on CPU clusters to localize, track and perform diffusion analysis on tens of thousands of individual split-GFP fusions in multiple worms. SM tracking in dCALM movies was performed by 2D Gaussian fitting of the point-spread function for each activated CD4-split-GFP or VDCC in each frame. The mean localization precision±s.d. for all individual fusion protein detected in CALM movies was determined by the method of Thompson *et al.*^59^ Trajectories were built by linking individual localized positions from one frame to the other, taking into account blinking statistics and local particle densities. Only the trajectories with at least three-step sizes were kept for ensemble and individual mean square displacement (MSD) analyses and for probability distribution of the displacement squared (PDSD) analyses.

For diffusion analyses, MSD and PDSD curves were computed^17^,^43^. For PDSD, computations were performed on the first ten observation time lags, a number sufficient to detect the confinement of CD4-split-GFP or VDCC on *C. elegans* muscles under our imaging conditions. In brief, for the first ten time lags *t*, each *Pr*^2^ curve was fitted with the general model:


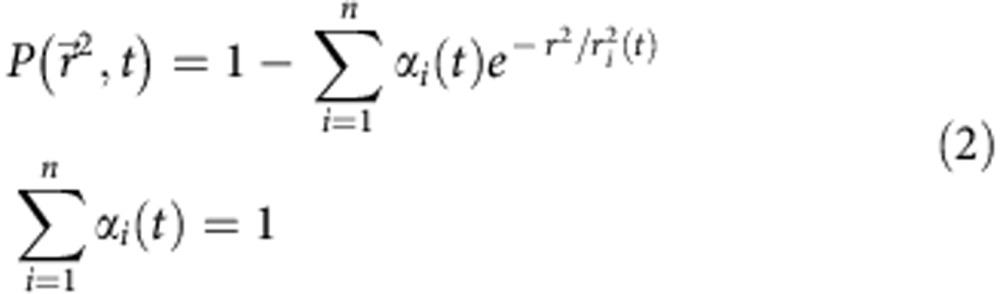


where the fitting coefficient *r*_*i*_^2^(t) and *α*_i_(*t*) are the square displacement and the fraction corresponding to *i* numbers of diffusion regimes at each time lag *t*, respectively. *Pr*^2^ distribution for CD4-split-GFP was best fitted with *i*=3 diffusion regimes, while *Pr*^2^ distribution for VDCC were fitted with *i*=2 regimes.

Error bars in MSD curves correspond to the s.e.m. Error bars for each *r*_*i*_^2^ in *r*_*i*_^2^(t) curves were determined using 
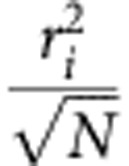
, where *N* represents the number of independent data points used to build each probability distribution functions^17^,^60^.

Diffusion coefficients were obtained by fitting MSD or *r*_*i*_^2^(t) curves using:

(i) an anomalous diffusion model:


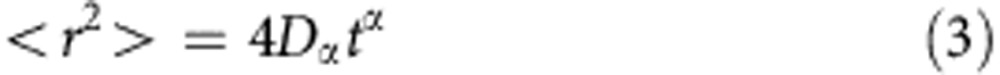


where *α* is the anomalous exponent and *D*_*α*_ is the time-dependent diffusion coefficient;

(ii) A free Brownian diffusion model with measurement error:


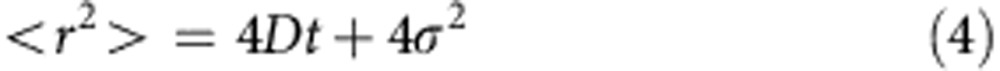


where *σ* is the position error and *D* is the diffusion coefficient;

or (iii) a circularly confined diffusion model with measurement error:


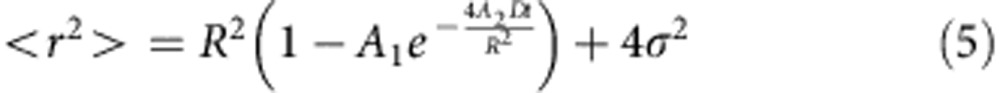


where *R* is the confinement radius, *σ* is the position error, *D* is the diffusion coefficient, *A*_1_=0.99 and *A*_2_=0.85 (ref. 43)^43^.

Fitting was performed using Origin software (OriginLab). When diffusion was found to be anomalous over the first ten time lags on *r*_*i*_^2^(t) curves, the linear part of the curves were fitted with the free Brownian diffusion model to assess short-scale diffusion coefficients and free diffusion distances. To build the distribution of diffusion coefficients for individual VDCC, the free Brownian diffusion model was fitted over the first three points of each individual MSD. All the diffusion coefficients D are reported in micrometre squared per second±s.d. of the fit value. Significant differences between diffusion coefficients were determined using F-tests.

Intensity time trace analyses on individual VDCC were performed by integrating the fluorescence intensity within a 3 × 3 pixel region centred on the point-spread function of each activated VDCC and for the whole length of acquired dCALM movies, including periods with no GFP signal before activation and after photobleaching. Background correction was performed by subtracting the fluorescence intensity at each time point from a similar 3 × 3 pixel region in the immediate vicinity of each individual VDCC. The slight differences in intensity between time traces are because of moderate variations in HILO excitation field uniformity when imaging different muscle cells or nerve cords in worms.

### Distribution pattern analyses of VDCC nanodomains

Ripley’s K function analyses and nearest-neighbour distance measurements were performed with the software SpPack^61^ after 2D Gaussian fitting of the nanodomain positions in sum intensity projection images of SM dCALM movies.

Ripley’s K analyses with edge corrections were performed on muscle cells for multiple regions of interest totaling 650–2,000 μm^2^ of the sarcolemma membrane in multiple worms for each condition. 95% confidence intervals for complete spatial randomness were obtained by averaging Monte Carlo simulations consisting of 500 replicates in multiple regions of interest with areas and nanodomain seeding numbers similar to that of the worm data.

For nearest-neighbour distance measurements, 1,100–2,500 nanodomains were analysed for each condition and data are reported as the mean nearest-neighbour distance in nanometre±s.e.m. A non-parametric Wilcoxon sign-rank statistical test was performed to assess significant differences between data sets.

## Author contributions

F.P. and J.-L. B. conceived and designed the experiments, H.Z., R.S., C.S., K.K.D., M.J. and F.P. performed experiments and analysed the data. H.Z., M.J. and J.-L.B. provided editorial input on the manuscript. F.P. wrote the manuscript.

## Additional information

**How to cite this article**: Zhan, H. *et al.* *In vivo* single-molecule imaging identifies altered dynamics of calcium channels in dystrophin-mutant *C. elegans*. *Nat. Commun.* 5:4974 doi: 10.1038/ncomms5974 (2014).

## Supplementary Material

Supplementary Information Supplementary Figures 1-7, Supplementary Table 1 and Supplementary Reference.

Supplementary Movie 1 Microinjection of synthetic M3 complementary peptides in the pseudocoelomic cavity of a live adult worm.

Supplementary Movie 2 Single molecule HILO dCALM imaging and tracking of activated CD4-split-GFP at the sarcolemma of a body-wall muscle cell in a live anesthetized C. elegans worm. Left panel: raw data. Right panel: Trajectories of individual activated CD4-split-GFP (green). Movie acquired at 100 ms per frame and played back at video rate.

Supplementary Movie 3 Single molecule HILO dCALM imaging of individual VDCC by activation of UNC-36-split-GFP at the sarcolemma of body-wall muscles in a live anesthetized C. elegans worm. The muscles twitch sporadically during imaging. Movie acquired at 80 ms per frame and played back at video rate.

Supplementary Movie 4 HILO dCALM imaging of two C. elegans worms expressing UNC-36-split-GFP and microinjected with a high concentration of complementary M3 peptides (left panel) or not microinjected (right panel). The objective lens is moved to focus at the muscle sarcolemma and the angle of the 488 nm excitation laser beam is changed to achieve HILO and low background detection of activated UNC-36-split-GFP on body-wall muscles. Activated and fluorescent UNC-36-split-GFP are only detected in the microinjected worm (left panel). Movie acquired at 80 ms per frame and played back at video rate.

Supplementary Movie 5 HILO dCALM imaging of VDCC in a normal, bead-immobilized worm where some muscles undergo active contractions (arrowhead). VDCC are strongly stabilized in nanodomains and at each contraction the inter-distance between nanodomains transiently decreases before increasing back during relaxation. This consecutive reductions and augmentations of VDCC nanodomains inter-distances suggest that the sarcolemma undergoes periodic deformations at each contraction/relaxation cycle, reminiscent of sarcolemma festooning. Movie acquired at 80 ms per frame and played back at video rate.

Supplementary Movie 6 HILO dCALM imaging of VDCC in resting and contracted muscles of normal (top) or dys-1 dystrophic worms (bottom). Bead-immobilized worms (resting muscles) display occasional body and muscle movements. Worms anesthetized with levamisole (contracted muscles) also display occasional muscle twitching. VDCC tracking was only performed between worm movements or muscle twitching. At the macroscale, no obvious differences in VDCC distribution and localization at the sarcolemma are observed between worms. Movie acquired at 80 ms per frame and played back at video rate.

## References

[b1] Lord S. J., Lee H. L. & Moerner W. E. Single-molecule spectroscopy and imaging of biomolecules in living cells. Anal. Chem. 82, 2192–2203 (2010). 20163145 10.1021/ac9024889 PMC2838489

[b2] Schaaf M. J.M. *et al.* Single-molecule microscopy reveals membrane microdomain organization of cells in a living vertebrate. Biophys. J. 97, 1206–1214 (2009). 19686669 10.1016/j.bpj.2009.05.044 PMC2726327

[b3] Shi X. *et al.* Determination of dissociation constants in living zebrafish embryos with single wavelength fluorescence cross-correlation spectroscopy. Biophys. J. 97, 678–686 (2009). 19619483 10.1016/j.bpj.2009.05.006 PMC2711317

[b4] Ries J., Yu S. R., Burkhardt M., Brand M. & Schwille P. Modular scanning FCS quantifies receptor-ligand interactions in living multicellular organisms. Nat. Methods 6, 643–U631 (2009). 19648917 10.1038/nmeth.1355

[b5] Petrasek Z. *et al.* Characterization of Protein Dynamics in Asymmetric Cell Division by Scanning Fluorescence Correlation Spectroscopy. Biophys. J. 95, 5476–5486 (2008). 18805921 10.1529/biophysj.108.135152 PMC2586573

[b6] Robin F. B., McFadden W. M., Yao B. & Munro E. M. Single-molecule analysis of cell surface dynamics in *Caenorhabditis elegans* embryos. Nat. Methods 11, 677–682 (2014). 24727651 10.1038/nmeth.2928 PMC4046709

[b7] Tokunaga M., Imamoto N. & Sakata-Sogawa K. Highly inclined thin illumination enables clear single-molecule imaging in cells. Nat. Methods 5, 159–161 (2008). 18176568 10.1038/nmeth1171

[b8] Ritter J. G., Veith R., Veenendaal A., Siebrasse J. P. & Kubitscheck U. Light sheet microscopy for single molecule tracking in living tissue. PLoS ONE 5, e11639 (2010). 20668517 10.1371/journal.pone.0011639 PMC2909143

[b9] Cella Zanacchi F. *et al.* Live-cell 3D super-resolution imaging in thick biological samples. Nat. Methods 8, 1047–1049 (2011). 21983925 10.1038/nmeth.1744

[b10] Stepanenko O. V. *et al.* Modern fluorescent proteins: from chromophore formation to novel intracellular applications. Biotechniques 51, 313 (2011). 22054544 10.2144/000113765 PMC4437206

[b11] Yano Y. & Matsuzaki K. Tag-probe labeling methods for live-cell imaging of membrane proteins. Biochim. Biophys. Acta 1788, 2124–2131 (2009). 19646952 10.1016/j.bbamem.2009.07.017

[b12] Pinaud F. & Dahan M. Targeting and imaging single biomolecules in living cells by complementation-activated light microscopy with split-fluorescent proteins. Proc. Natl Acad. Sci. USA 108, E201–E210 (2011). 21606345 10.1073/pnas.1101929108 PMC3116416

[b13] Mathews E. A. *et al.* Critical residues of the *Caenorhabditis elegans* unc-2 voltage-gated calcium channel that affect behavioral and physiological properties. J. Neurosci. 23, 6537–6545 (2003). 12878695 10.1523/JNEUROSCI.23-16-06537.2003 PMC6740628

[b14] Gao S. & Zhen M. Action potentials drive body wall muscle contractions in *Caenorhabditis elegans*. Proc. Natl Acad. Sci. USA 108, 2557–2562 (2011). 21248227 10.1073/pnas.1012346108 PMC3038695

[b15] Sancar F. *et al.* The dystrophin-associated protein complex maintains muscle excitability by regulating Ca2+-dependent K+ (BK) channel localization. J. Biol. Chem. 286, 33501–33510 (2011). 21795674 10.1074/jbc.M111.227678 PMC3190934

[b16] Feinberg E. H. *et al.* GFP reconstitution across synaptic partners (GRASP) defines cell contacts and Synapses in living nervous systems. Neuron 57, 353–363 (2008). 18255029 10.1016/j.neuron.2007.11.030

[b17] Schutz G. J., Schindler H. & Schmidt T. Single-molecule microscopy on model membranes reveals anomalous diffusion. Biophys. J. 73, 1073–1080 (1997). 9251823 10.1016/S0006-3495(97)78139-6 PMC1181003

[b18] Simson R. *et al.* Structural mosaicism on the submicron scale in the plasma membrane. Biophys. J. 74, 297–308 (1998). 9449330 10.1016/S0006-3495(98)77787-2 PMC1299382

[b19] Stuhmer W. & Almers W. Photobleaching through glass micropipettes: sodium channels without lateral mobility in the sarcolemma of frog skeletal muscle. Proc. Natl Acad. Sci. USA 79, 946–950 (1982). 6278504 10.1073/pnas.79.3.946 PMC345870

[b20] Saheki Y. & Bargmann C. I. Presynaptic CaV2 calcium channel traffic requires CALF-1 and the alpha(2)delta subunit UNC-36. Nat. Neurosci. 12, 1257–1265 (2009). 19718034 10.1038/nn.2383 PMC2805665

[b21] Laine V., Frokjaer-Jensen C., Couchoux H. & Jospin M. The alpha 1 Subunit EGL-19, the alpha 2/delta subunit UNC-36, and the beta subunit CCB-1 underlie voltage-dependent calcium currents in *Caenorhabditis elegans* striated muscle. J. Biol. Chem. 286, 36180–36187 (2011). 21878625 10.1074/jbc.M111.256149 PMC3196126

[b22] Frokjaer-Jensen C. *et al.* Single-copy insertion of transgenes in *Caenorhabditis elegans*. Nat. Genet. 40, 1375–1383 (2008). 18953339 10.1038/ng.248 PMC2749959

[b23] Davies A. *et al.* The alpha2delta subunits of voltage-gated calcium channels form GPI-anchored proteins, a posttranslational modification essential for function. Proc. Natl Acad. Sci. USA 107, 1654–1659 (2010). 20080692 10.1073/pnas.0908735107 PMC2824380

[b24] Altun Z. F. & Hall D. H. Muscle system, introduction, in WormAtlas (2009).

[b25] Street S. F. Lateral transmission of tension in frog myofibers: a myofibrillar network and transverse cytoskeletal connections are possible transmitters. J. Cell Physiol. 114, 346–364 (1983). 6601109 10.1002/jcp.1041140314

[b26] Ervasti J. M. Costameres: the Achilles' heel of Herculean muscle. J. Biol. Chem. 278, 13591–13594 (2003). 12556452 10.1074/jbc.R200021200

[b27] Le Rumeur E., Winder S. J. & Hubert J. F. Dystrophin: more than just the sum of its parts. Biochim. Biophys. Acta 1804, 1713–1722 (2010). 20472103 10.1016/j.bbapap.2010.05.001

[b28] Lansman J. B. & Franco A. What does dystrophin do in normal muscle. Journal of Muscle Research and Cell Motility 12, 409–411 (1991). 1658040 10.1007/BF01738325

[b29] Kumar A., Khandelwal N., Malya R., Reid M. B. & Boriek A. M. Loss of dystrophin causes aberrant mechanotransduction in skeletal muscle fibers. FASEB J. 18, 102–113 (2004). 14718391 10.1096/fj.03-0453com

[b30] Zsnagy I., Zhang X., Kitani K. & Nonomura Y. The influence of dystrophin on lateral diffusion of proteins in sarcolemma of L-185 and C2 myoblasts and mature striated-muscle cells of rats and mice, as measured by FRAP technique. Biochim. Biophys. Res. Commun. 215, 67–74 (1995). 10.1006/bbrc.1995.2434 7575626

[b31] Sarkis J. *et al.* Resisting sarcolemmal rupture: dystrophin repeats increase membrane-actin stiffness. FASEB J. 27, 359–367 (2013). 23033320 10.1096/fj.12-208967

[b32] Dickinson D. J., Ward J. D., Reiner D. J. & Goldstein B. Engineering the *Caenorhabditis elegans* genome using Cas9-triggered homologous recombination. Nat. Methods 10, 1028 (2013). 23995389 10.1038/nmeth.2641 PMC3905680

[b33] Robert V. J. & Bessereau J. L. Genome engineering by transgene-instructed gene conversion in *C. elegans*. Methods Cell Biol. 106, 65–88 (2011). 22118274 10.1016/B978-0-12-544172-8.00003-7

[b34] Best J. M. & Kamp T. J. Different subcellular populations of L-type Ca2+ channels exhibit unique regulation and functional roles in cardiomyocytes. J. Mol. Cell. Cardiol. 52, 376–387 (2012). 21888911 10.1016/j.yjmcc.2011.08.014 PMC3264751

[b35] Balijepalli R. C., Foell J. D., Hall D. D., Hell J. W. & Kamp T. J. Localization of cardiac L-type Ca(2+) channels to a caveolar macromolecular signaling complex is required for beta(2)-adrenergic regulation. Proc. Natl Acad. Sci. USA 103, 7500–7505 (2006). 16648270 10.1073/pnas.0503465103 PMC1564282

[b36] Suzuki Y., Yamamura H., Ohya S. & Imaizumi Y. Caveolin-1 facilitates the direct coupling between large conductance Ca2+-activated K+ (BKCa) and Cav1.2 Ca2+ channels and their clustering to regulate membrane excitability in vascular myocytes. J. Biol. Chem. 288, 36750–36761 (2013). 24202214 10.1074/jbc.M113.511485 PMC3868784

[b37] Tang Z. *et al.* Identification, sequence, and expression of an invertebrate caveolin gene family from the nematode *Caenorhabditis elegans*. Implications for the molecular evolution of mammalian caveolin genes. J. Biol. Chem. 272, 2437–2445 (1997). 8999956 10.1074/jbc.272.4.2437

[b38] Kirkham M. *et al.* Evolutionary analysis and molecular dissection of caveola biogenesis. J. Cell Sci. 121, 2075–2086 (2008). 18505796 10.1242/jcs.024588

[b39] Head B. P. & Insel P. A. Do caveolins regulate cells by actions outside of caveolae? Trends Cell Biol. 17, 51–57 (2007). 17150359 10.1016/j.tcb.2006.11.008

[b40] Lingwood D. & Simons K. Lipid Rafts as a membrane-organizing principle. Science 327, 46–50 (2010). 20044567 10.1126/science.1174621

[b41] Rao W., Isaac R. E. & Keen J. N. An analysis of the *Caenorhabditis elegans* lipid raft proteome using geLC-MS/MS. J. Proteomics 74, 242–253 (2011). 21070894 10.1016/j.jprot.2010.11.001

[b42] Davies A. *et al.* The calcium channel alpha2delta-2 subunit partitions with CaV2.1 into lipid rafts in cerebellum: implications for localization and function. J. Neurosci. 26, 8748–8757 (2006). 16928863 10.1523/JNEUROSCI.2764-06.2006 PMC6674382

[b43] Pinaud F. *et al.* Dynamic partitioning of a glycosyl-phosphatidylinositol-anchored protein in glycosphingolipid-rich microdomains imaged by single-quantum dot tracking. Traffic 10, 691–712 (2009). 19416475 10.1111/j.1600-0854.2009.00902.x PMC2766537

[b44] Tadross M. R., Tsien R. W. & Yue D. T. Ca2+ channel nanodomains boost local Ca2+ amplitude. Proc. Natl Acad. Sci. USA 110, 15794–15799 (2013). 24019485 10.1073/pnas.1313898110 PMC3785779

[b45] Maryon E. B., Saari B. & Anderson P. Muscle-specific functions of ryanodine receptor channels in *Caenorhabditis elegans*. J. Cell Sci. 111, (Pt 19): 2885–2895 (1998). 9730981 10.1242/jcs.111.19.2885

[b46] Maryon E. B., Coronado R. & Anderson P. unc-68 encodes a ryanodine receptor involved in regulating *C. elegans* body-wall muscle contraction. J. Cell biol. 134, 885–893 (1996). 8769414 10.1083/jcb.134.4.885 PMC2120954

[b47] Robertson A. P., Clark C. L. & Martin R. J. Levamisole and ryanodine receptors. I: A contraction study in *Ascaris suum*. Mol. Biochem. Parasitol. 171, 1–7 (2010). 20064566 10.1016/j.molbiopara.2009.12.007 PMC2838958

[b48] Berkefeld H. *et al.* BKCa-Cav channel complexes mediate rapid and localized Ca2+-activated K+ signaling. Science 314, 615–620 (2006). 17068255 10.1126/science.1132915

[b49] Kim H. *et al.* The dystrophin complex controls bk channel localization and muscle activity in *Caenorhabditis elegans*. PLoS Genet. 5, e1000780 (2009). 20019812 10.1371/journal.pgen.1000780 PMC2788698

[b50] Bessou C., Giugia J. B., Franks C. J., Holden-Dye L. & Segalat L. Mutations in the *Caenorhabditis elegans* dystrophin-like gene dys-1 lead to hyperactivity and suggest a link with cholinergic transmission. Neurogenetics 2, 61–72 (1998). 9933302 10.1007/s100480050053

[b51] Allard B. Sarcolemmal ion channels in dystrophin-deficient skeletal muscle fibres. J. Muscle Res. Cell Motil. 27, 367–373 (2006). 16874448 10.1007/s10974-006-9083-4

[b52] Berchtold M. W., Brinkmeier H. & Muntener M. Calcium ion in skeletal muscle: Its crucial role for muscle function, plasticity, and disease. Physiol. Rev. 80, 1215–1265 (2000). 10893434 10.1152/physrev.2000.80.3.1215

[b53] Laine V., Frokjaer-Jensen C., Couchoux H. & Jospin M. The alpha1 subunit EGL-19, the alpha2/delta subunit UNC-36, and the beta subunit CCB-1 underlie voltage-dependent calcium currents in *Caenorhabditis elegans* striated muscle. J. Biol. Chem. 286, 36180–36187 (2011). 21878625 10.1074/jbc.M111.256149 PMC3196126

[b54] Gally C. & Bessereau J. L. GABA is dispensable for the formation of junctional GABA receptor clusters in *Caenorhabditis elegans*. J. Neurosci. 23, 2591–2599 (2003). 12684444 10.1523/JNEUROSCI.23-07-02591.2003 PMC6742079

[b55] Francis G. R. & Waterston R. H. Muscle organization in *Caenorhabditis elegans*: localization of proteins implicated in thin filament attachment and I-band organization. J. Cell Biol. 101, 1532–1549 (1985). 2413045 10.1083/jcb.101.4.1532 PMC2113919

[b56] Gottschalk A. & Schafer W. R. Visualization of integral and peripheral cell surface proteins in live *Caenorhabditis elegans*. J. Neurosci.Methods 154, 68–79 (2006). 16466809 10.1016/j.jneumeth.2005.11.016

[b57] Kim E., Sun L., Gabel C. V. & Fang-Yen C. Long-term imaging of *Caenorhabditis elegans* using nanoparticle-mediated immobilization. PLoS ONE 8, e53419 (2013). 23301069 10.1371/journal.pone.0053419 PMC3536676

[b58] Serge A., Bertaux N., Rigneault H. & Marguet D. Dynamic multiple-target tracing to probe spatiotemporal cartography of cell membranes. Nat. Methods 5, 687–694 (2008). 18604216 10.1038/nmeth.1233

[b59] Thompson R. E., Larson D. R. & Webb W. W. Precise nanometer localization analysis for individual fluorescent probes. Biophys. J. 82, 2775–2783 (2002). 11964263 10.1016/S0006-3495(02)75618-X PMC1302065

[b60] Qian H., Sheetz M. P. & Elson E. L. Single-particle tracking - analysis of diffusion and flow in 2-dimensional systems. Biophys. J. 60, 910–921 (1991). 1742458 10.1016/S0006-3495(91)82125-7 PMC1260142

[b61] Perry G. L.W. SpPack: spatial point pattern analysis in Excel using Visual Basic for Applications (VBA). Environ. Model. Software 19, 559–569 (2004).

[b62] Moerman D. G. & Williams B. D. Sarcomere assembly in *C. elegans* muscle. WormBook : the online review of C. elegans biology 1–16 (2006). 18050483 10.1895/wormbook.1.81.1 PMC4781162

