# Supplementary material for: In vivo single-molecule imaging identifies altered dynamics of calcium channels in dystrophin-mutant C. elegans
Source: Nat Commun. 2014 Sep 18;5:4974. doi: 10.1038/ncomms5974 (PMC4199201; doi:10.1038/ncomms5974)
Supplement: Supplementary Information — Supplementary Figures 1-7, Supplementary Table 1 and Supplementary Reference. (PDF 1000 kb) [file 41467_2014_BFncomms5974_MOESM1208_ESM.pdf]

Supplementary Figures and Tables

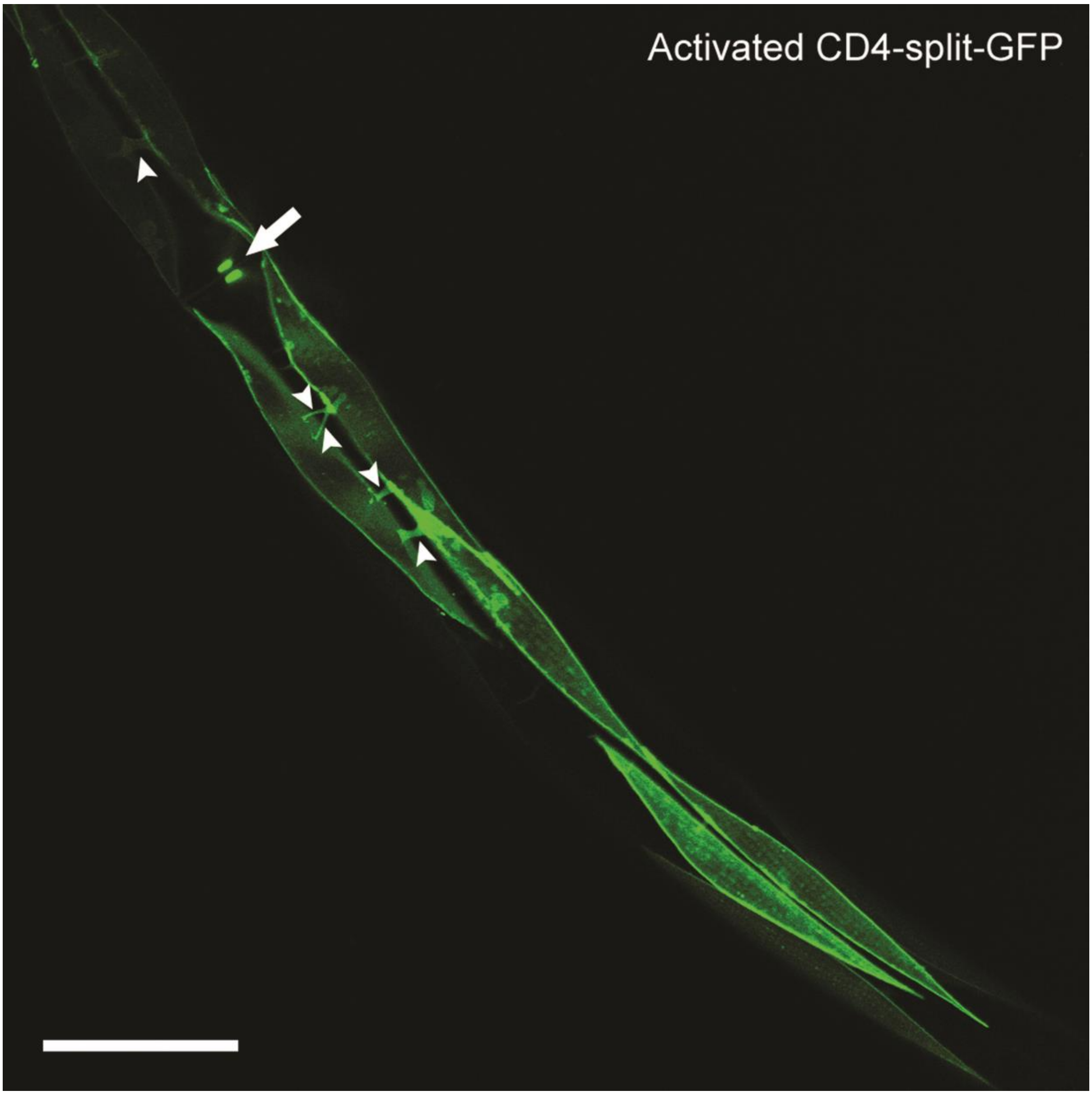

Supplementary Fig. 1: dCALM confocal imaging of CD4-split-GFP activated at the surface of body-wall and vulval muscles (arrow) in live *C. elegans*. CD4-split-GFP is also activated on muscle arms (arrow-heads). Scale bar: 50 µm.

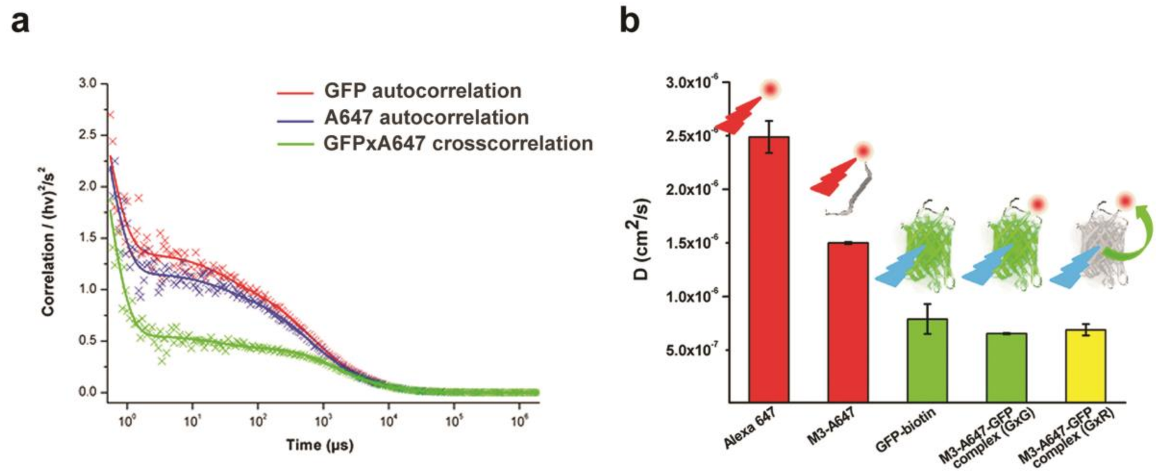

Supplementary Fig. 2: Fluorescence auto- and cross-correlation spectroscopy (FCS/FCCS) of M3-A647 irreversible binding to recombinant split-GFP. 1:1 irreversible binding between recombinant split-GFP and fluorescent M3-A647 complementary peptides was tested on complemented M3-A647-GFP complex purified by size exclusion chromatography and studied at single molecule concentration (250 pM) by FCS and FCCS. a) Auto and cross-correlation curves of M3-A647-GFP complex at 488 nm excitation and 520 nm emission (GFP signal only, red), at 633 nm excitation and 670 nm emission (M3-A647 signal only, blue) and at 488 nm excitation and simultaneous detection at 520 nm and 670 nm (spFRET signal only, green). b) Translational diffusion coefficients obtained from FCS and FCCS analysis. M3-A647-GFP complex (spFRET signal, yellow) freely diffuse in solution with a coefficient of $6.89 \pm 0.53 \times 10^{-7}$ cm^2/s similar to the published value for EGFP[1] and similar to that of recombinant split-GFP complemented with non-fluorescent M3-biotin peptides (green). This diffusion value is significantly slower than that of free A647 or that of free M3-A647 peptides indicating that M3-A647 remains strongly bound in M3-A647-GFP complex even at picomolar concentrations. The detection of spFRET between activated GFP and A647 at picomolar concentrations of M3-A647-GFP complex confirms the 1:1 stochiometry and irreversible attachment of complementary peptides upon activation of recombinant split-GFP.

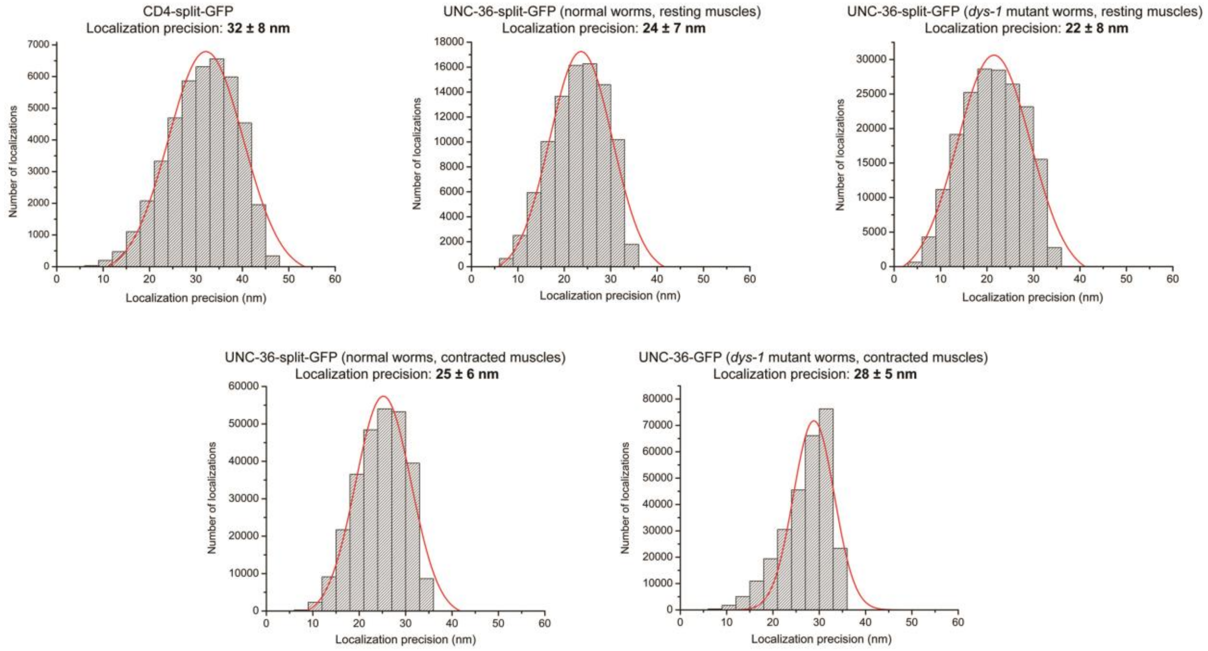

Supplementary Fig. 3: Mean localization precision (± standard deviation of the mean) of individual CD4-split-GFP and UNC-36-split-GFP at the sarcolemma of muscle cells in live *C. elegans* worms, during SM dCALM tracking.

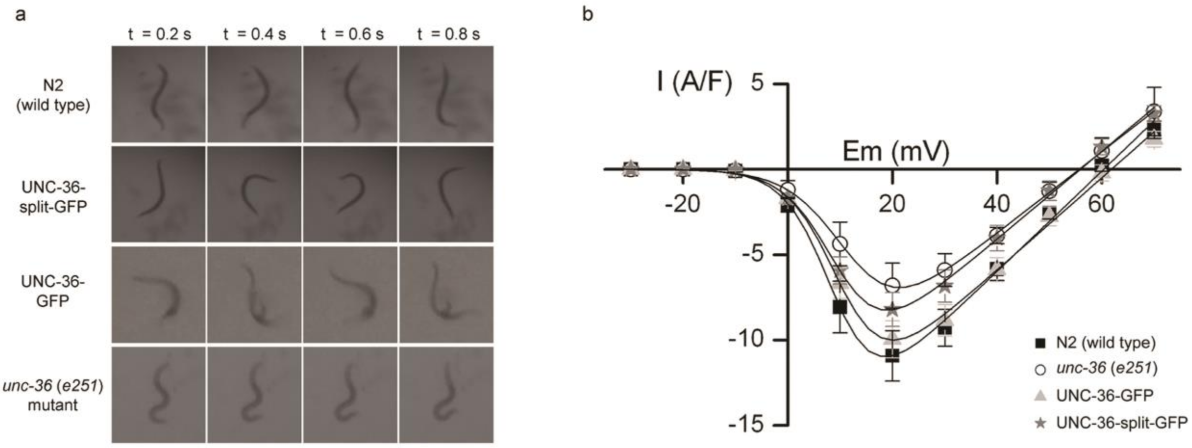

Supplementary Fig. 4: Rescue of UNC-36 activity and VDCC function in *unc-36* (*e251*) null allele mutant worms by single copy insertion of UNC-36-split-GFP or UNC-36-GFP via *Mos*SCI. a) Trashing assay in M9 media at 20^{0}C. The impaired motility of *unc-36* (*e251*) mutant worms is fully rescued with UNC-36-split-GFP or UNC-36-GFP expression and the frequency of body bends is undistinguishable for that of wild type N2 worms. b) Mean current-voltage relationships (± standard error of the mean) of VDCC established for the peak currents in N2 wild-type (n=8), *unc-36(e251)* (n=7), UNC-36-GFP (n=7) and UNC-36-split-GFP (n=8) worms. Current-voltage relationships were fitted by equation (1) to obtained $E_{0.5}$ values from Fig. 3.

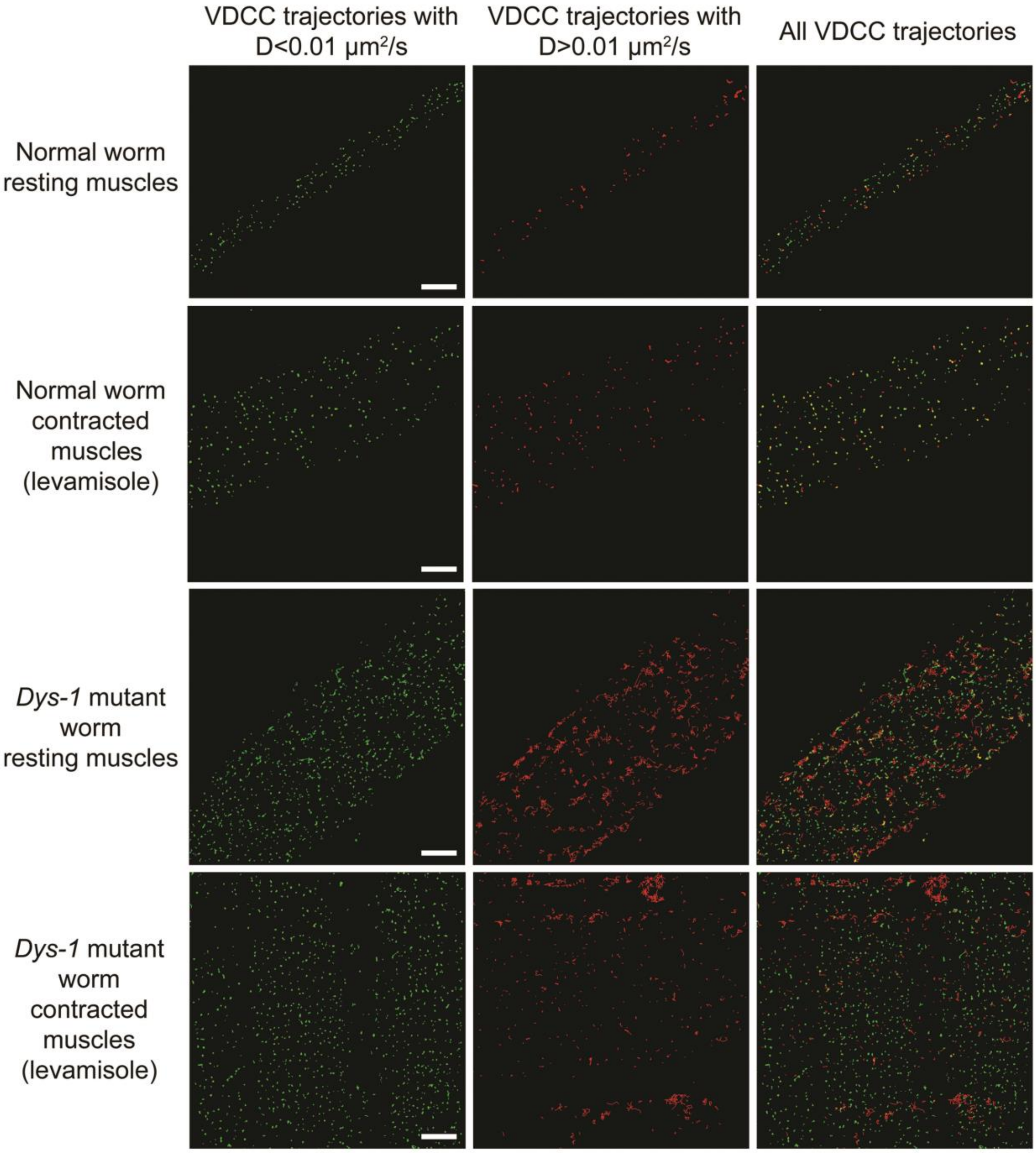

Supplementary Fig. 5: Representative sarcolemma diffusion maps of VDCC trajectories with diffusion coefficients below (slow VDCC, green) or above (fast VDCC, red) 0.02 $\mu m^2/s$. Diffusion coefficients were determined from MSD analysis of individual trajectories by fitting the first three non-zero MSD points with a pure Brownian diffusion model (free diffusion). The slow and fast VDCC populations are distributed independently at the plasma membrane of both resting and contracted muscles in normal and dystrophic worms. Differences in the width of muscle cells result from varying orientation of the worms. Scale bars: 10 μm.

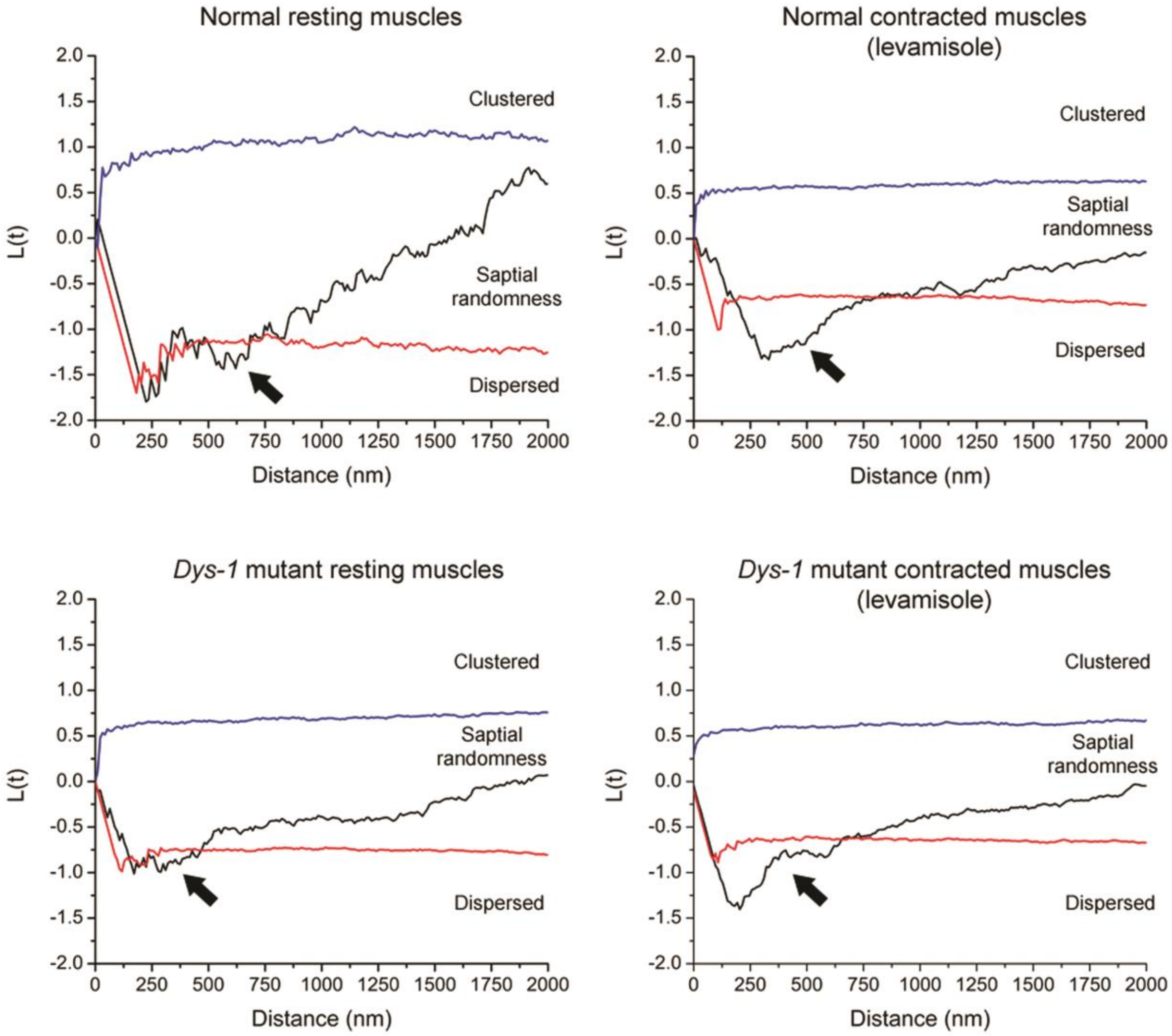

Supplementary Fig. 6: Ripley's K space clustering analysis of VDCC nanodomains distribution in resting or contracted muscle sarcolemma of normal and dystrophic *dys-1 C. elegans* worms. The VDCC nanodomains are not clustered at the sarcomere surface but are significantly more dispersed than expected for complete spatial randomness at length scales of 250-750 nm, for all conditions (arrow). Blue and red lines represent the 95% confidence intervals for complete spatial randomness determined using 500 replicates of a random distribution for areas similar in size to that analyzed on muscle cells.

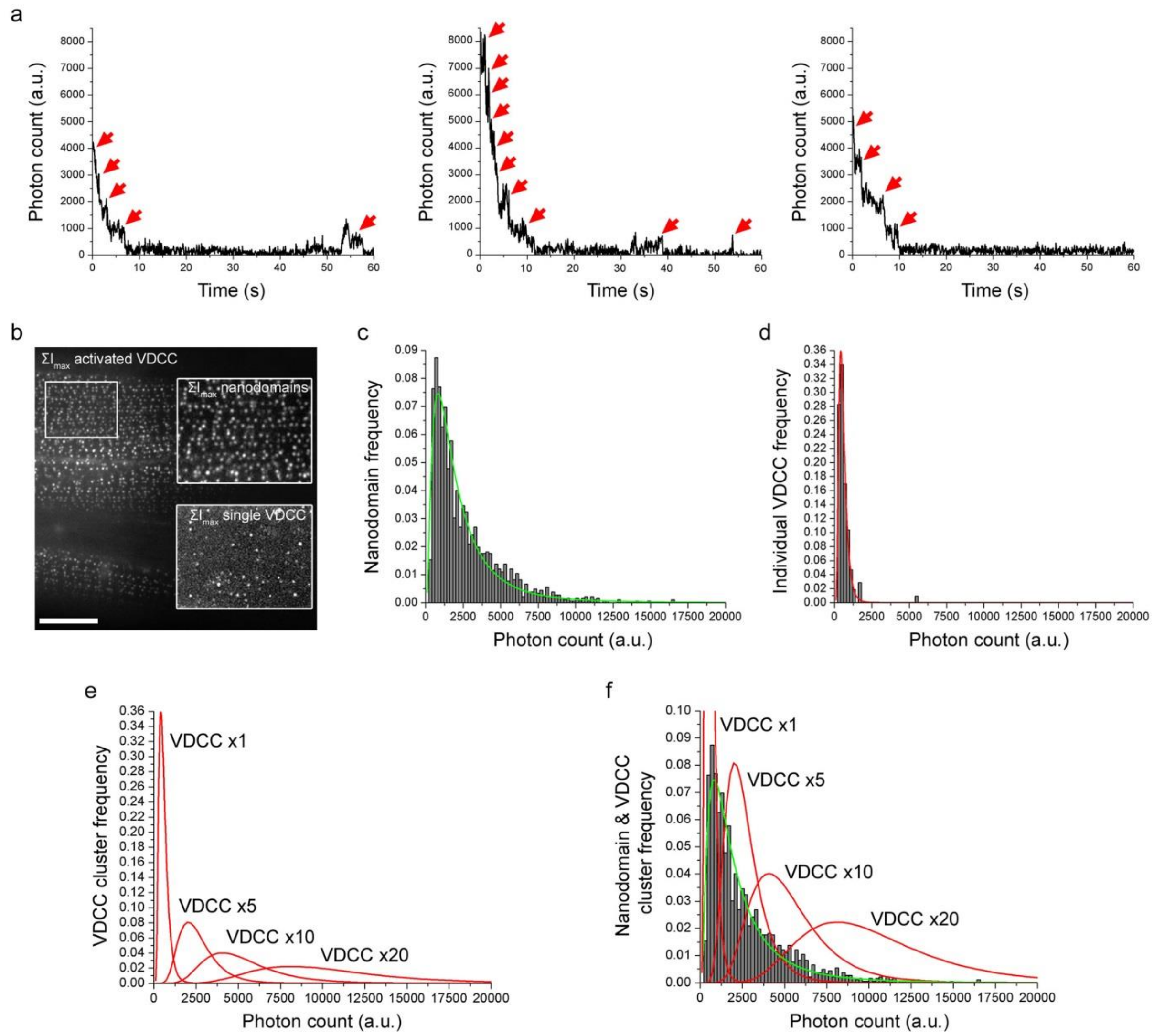

Supplementary Fig. 7: Quantitative analyses of the number of VDCC in individual nanodomains at the sarcolemma. a) Background corrected intensity time traces for three individual nanodomains in worms imaged by HILO after injection of a high concentration of M3 peptides (5mM) to activate as many VDCC as possible in each nanodomain. Step-wise changes in fluorescence intensity of the nanodomains stemming from individual VDCC photobleaching or activation are indicated by red arrows. Each nanodomains display different numbers of VDCC because of the stochastic activation of VDCC by CALM. At least 10 VDCC can be detected in some nanodomains. b) Maximum intensity projection image of activated VDCC on muscle cells for a worm injected with high concentration of M3 peptides (5mM). Pixels in this maximum intensity projection image contain the maximum value over all images in a HILO movie at a particular pixel location and therefore represent the cumulative intensity of all activated VDCC in a particular nanodomain before any VDCC is photobleached. Insets: Region of interest (white square) showing the maximum intensity projection image of the entire HILO movie (top) and that of the last 500 frames of the same movie (bottom), a time frame at which all VDCC have already been photobleached and only a few individual VDCC are detected upon activation by residual M3 peptides circulating in the worm body. Scale bar: 10 µm. c) Maximum intensity distribution for 1820 nanodomains at the muscle sarcolemma of worms injected with high concentration of M3 peptides. The distribution is Lognormal (green fit) and wide because of variations in the number of activated VDCC in each nanodomain (CALM stochasticity) and because of

inherent variations in HILO excitation field uniformity or muscle cell orientation. d) Maximum intensity distribution for 107 individual sarcolemmal VDCC detected in the last few hundred frames of same HILO movies used in (c). The distribution is Lognormal (red fit), but much narrower than for nanodomains in (c). e) Simulated maximum intensity distributions for various VDCC clusters. The maximum fluorescence intensity value for each 107 VDCC in (d) was multiplied by a factor x5, x10 or x20 to simulate the intensity distribution of VDCC clusters containing 5, 10 or 20 VDCC respectively. f) Overlay of the maximum intensity distribution for individual nanodomains (grey bars and green fit) and for simulated VDCC clusters (red fits). The nanodomain intensity distribution is much larger than that of individual VDCC, indicating that most nanodomains contain more than 1 VDCC when activating as many VDCC as possible with the injection of high concentrations of M3 peptides. The nanodomain intensity distribution also shows significant overlap with the expected intensity distribution of VDCC clusters containing 5 or 10 VDCC but has limited overlap with that expected for 20 VDCC clusters. In the light of these data and those in (a) we conclude that, although we cannot precisely determine the exact number of VDCC per nanodomain, some nanodomains contain at least 10 VDCC.

Supplementary Table 1: Confinement sizes and free diffusion lengths of VDCC at the sarcolemma of *C. elegans* worms.

**Worms**	**Muscle state**	**VDCC population**	**Confinement size (nm ± std. error)**	**Free diffusion length (nm ± std. error)**
Normal	Resting	Slow	82 ± 2	
		Fast		230 ± 8
	Contracted (Levamisole)	Slow	No confinement	
		Fast		224 ± 9
dys-1 mutant	Resting	Slow	58 ± 2	
		Fast		303 ± 10
	Contracted (Levamisole)	Slow	52 ± 2	
		Fast		281 ± 6

Supplementary References

1. Hess, S.T., Sheets, E.D., Wagenknecht-Wiesner, A. & Heikal, A.A. Quantitative analysis of the fluorescence properties of intrinsically fluorescent proteins in living cells. *Biophysical Journal* 85, 2566-2580 (2003).
